# The invisible costs of obstructive sleep apnea (OSA): Systematic review and cost-of-illness analysis

**DOI:** 10.1371/journal.pone.0268677

**Published:** 2022-05-20

**Authors:** Ludovica Borsoi, Patrizio Armeni, Gleb Donin, Francesco Costa, Luigi Ferini-Strambi

**Affiliations:** 1 SDA Bocconi School of Management, Centre for Research on Health and Social Care Management (CERGAS), Milan, Italy; 2 Department of Biomedical Technology, Czech Technical University in Prague, Kladno, Czech Republic; 3 Faculty of Psychology, Vita-Salute San Raffaele University, Milan, Italy; Kaohsuing Medical University Hospital, TAIWAN

## Abstract

**Background:**

Obstructive sleep apnea (OSA) is a risk factor for several diseases and is correlated with other non-medical consequences that increase the disease’s clinical and economic burden. However, OSA’s impact is highly underestimated, also due to substantial diagnosis gaps.

**Objective:**

This study aims at assessing the economic burden of OSA in the adult population in Italy by performing a cost-of-illness analysis with a societal perspective. In particular, we aimed at estimating the magnitude of the burden caused by conditions for which OSA is a proven risk factor.

**Methods:**

A systematic literature review on systematic reviews and meta-analyses, integrated by expert opinion, was performed to identify all clinical and non-clinical conditions significantly influenced by OSA. Using the Population Attributable Fraction methodology, a portion of their prevalence and costs was attributed to OSA. The total economic burden of OSA for the society was estimated by summing the costs of each condition influenced by the disease, the costs due to OSA’s diagnosis and treatment and the economic value of quality of life lost due to OSA’s undertreatment.

**Results:**

Twenty-six clinical (e.g., diabetes) and non-clinical (e.g., car accidents) conditions were found to be significantly influenced by OSA, contributing to an economic burden ranging from €10.7 to €32.0 billion/year in Italy. The cost of impaired quality of life due to OSA undertreatment is between €2.8 and €9.0 billion/year. These costs are substantially higher than those currently borne to diagnose and treat OSA (€234 million/year).

**Conclusions:**

This study demonstrates that the economic burden due to OSA is substantial, also due to low diagnosis and treatment rates. Providing reliable estimates of the economic impact of OSA at a societal level may increase awareness of the disease burden and help to guide evidence-based policies and prioritisation for healthcare, ultimately ensuring appropriate diagnostic and therapeutic pathways for patients.

## Introduction

Obstructive sleep apnea (OSA) is a disorder characterized by episodic cessation of breathing due to repeated upper airway partial (hypopnea) or total (apnea) obstructions [1, 2]. These events lead to activation of the sympathetic nervous system, sleep fragmentation, intermittent hypoxemia and, in the case of syndrome (OSAS), to excessive daytime sleepiness [3, 4]. Diagnosis of OSA usually requires overnight laboratory polysomnography in order to detect the frequency of disordered breathing events [5]. OSA’s severity is measured through the number of apnea and hypopnea events per hour of sleep (apnea-hypopnea index, AHI), which can be more than 30 in its severe form [6, 7]. The attention towards the disorder is increasing as several studies found that OSA is a risk factor for a substantial number of clinical conditions in adults, including diabetes [8–10], cancer [11, 12], cardiovascular [13, 14] and cerebrovascular diseases [15–17]. Moreover, OSA and its syndrome are associated with decreased quality of life (QoL) [18–20], impaired work performance [21–23] and increased risk of road traffic accidents [24, 25]. According to several population-based studies, prevalence of OSA is relatively high, especially among men [26–31], although methodological differences and difficulties in characterizing this disorder yielded to variability in prevalence estimates [32, 33]. Despite the prevalence of this condition, OSA is frequently undiagnosed, either because patients do not regard their symptoms (e.g., snoring, excessive daytime sleepiness) as the presence of a disorder, thus not seeking medical consultation, or because primary care physicians are often unable to recognize OSA signs and symptoms [5, 34], leading to a potential underestimation of the disease burden and to consequent undertreatment [35, 36]. Therapy with continuous positive airway pressure (CPAP) represents the gold standard for the treatment of OSA [37, 38]. When adherence is optimal, CPAP has been demonstrated to reduce symptoms, the possible sequelae of the disease and to improve self-reported health status [39–42]. As OSA and its related syndrome have been demonstrated to influence the onset of several health conditions and other non-clinical consequences, their low diagnosis and treatment rates are likely to result in increased clinical and economic burden. Several studies have estimated that OSA is associated with substantial economic costs, especially if untreated [43–45]. However, to date there is not available evidence for Italy. The present study aims at assessing the societal economic burden associated with OSA in the adult population in Italy by performing a cost-of-illness (COI) analysis based on a systematic literature review and population attributable fraction methodology. In particular, we aimed at estimating the proportion of the cost-of-illness of conditions for which OSA is a proven risk factor that can be attributed to OSA itself. As in the literature there is not always a clear and consistent distinction between OSA and OSAS, we focused our analyses on the broadest definition of the disease (i.e., OSA). Providing reliable estimates of the economic impact of OSA at a societal level may increase awareness of the disease burden and help to guide evidence-based policies and prioritisation for healthcare in Italy, ultimately ensuring appropriate diagnostic and therapeutic pathways for patients.

## Materials and methods

A retrospective, prevalence-based COI study with a societal perspective and a one-year time-horizon was conducted to assess the economic burden of OSA and its syndrome for the Italian adult population.

### Estimation of OSA prevalence

A scoping review of the literature, both grey and peer-reviewed, was performed in order to establish the prevalence of OSA in Italy among adult population (aged 15–74 years). Findings from the literature were discussed with clinical experts in order to assess their reliability and generalizability to the Italian context. Moreover, on the basis of the data provided by the Italian association of apneic patients (Associazione Apnoici Italiani Onlus) and expert opinion, we estimated the number of patients currently diagnosed and treated in Italy, in order to assess the extent of underdiagnosis and undertreatment in the national context.

### Identification of conditions associated with OSA

A systematic literature review was carried out in order to identify the clinical and non-clinical conditions that have been demonstrated to be influenced by OSA in the adult population. The PRISMA guideline was used in developing this review [46]. The search was performed on PubMed according to a detailed search strategy and limited to studies providing the highest level of evidence, namely systematic reviews and meta-analyses (S1 Table). We decided to perform a “systematic review of systematic reviews” [47] since this approach is recommended when the literature is extremely heterogeneous in terms of methods, definitions and results, as in the case of OSA. The search was carried out on November 19^th^, 2018, and was updated on May 13^th^, 2021. The screening and selection of titles and abstracts first and full-texts later were conducted by two researchers in parallel on the basis of pre-defined exclusion criteria (S2 Table). Disagreements between reviewers on study inclusion were solved by consensus or by the decision of a third independent reviewer. The references and citations of the full-texts included were reviewed for additional articles according to a snowballing approach in order to ensure exhaustiveness of the review. Ultimately, studies were included if they provided quantitative evidence on the influence of OSA and its syndrome on other clinical and non-clinical conditions in the adult population. From the selected studies, the following data were extracted according to a predefined template: authors and year of publication; condition investigated (e.g., diabetes); OSA’s severity (i.e., mild, moderate, severe, overall); reference population (i.e., men, women, all); association measure (i.e., relative risk, hazard ratio, odds ratio); magnitude of association (mean value and 95% confidence intervals—CIs); statistical significance of the association (i.e., p-value). Data on magnitude of association (mean values) were used to calculate the proportion of conditions’ epidemiological burden influenced by OSA (see next section) and 95% CIs were used for sensitivity analysis. The exclusion criteria adopted and the results of the systematic review were discussed within a research board composed of health economists and clinicians specialized in different disciplines strongly related to OSA (i.e., neurology, endocrinology, internal medicine with cardiology specialization, gastroenterology). The aim of this phase was to ensure that the conditions retrieved through the review were significant from a clinical standpoint. Ultimately, only conditions for which a statistically significant association with OSA was found (i.e., p-value<0.05) and judged clinically meaningful by experts were included in the next steps of the analysis.

### Estimation of conditions’ burden influenced by OSA: Population attributable fraction

An extensive review of both published and grey literature was performed to collect Italian epidemiological data of conditions included. In case we could not retrieve an epidemiological study for Italy, we searched for studies referred to other countries. In case more than one study was available for the same condition, we considered the one providing most up-to-date estimates. When prevalence rates were reported, the total number of prevalent cases was derived using data on Italian population by age and sex provided by the Italian National Institute of Statistics for 2018 (Istat) [48].

In order to estimate the proportion of conditions’ epidemiological burden influenced by OSA and its syndrome, a Population Attributable Fraction (PAF) methodology was applied. The PAF can be defined as the proportional reduction in average disease risk that would be achieved by eliminating the exposure to a risk factor [49, 50]. The PAF allows to estimate the amount of disease burden caused by a certain risk factor. In the literature, there are different approaches to estimate PAF. In the present analysis, we chose the approach that was deemed more suitable according to the association data available (i.e., relative risk, odds ratio or hazard ratio). In particular, we used the formula proposed by Levin (1953) [51] when the measure of association provided was relative risk (RR):

$$PAF=\frac{p_{\left(E\right)}(RR-1)}{p_{\left(E\right)}\left(RR-1\right)+1}$$

where p_(E)_ is the prevalence of OSA; RR is relative risk.

It is important to highlight that Levin’s approach could lead to overestimation of PAF when the measure of association provided is adjusted RR [49]. However, studies included did not provide sufficient data to use alternative approaches, suitable in the presence of confounding, therefore we used Levin’s formula for both unadjusted and adjusted RR.

When the measure of association was hazard ratio (HR), a variant of the Levin’s formula was considered [52]:

$$PAF=\frac{p_{\left(E\right)}(HR(t)-1)}{p_{\left(E\right)}\left(HR(t)-1\right)+1}$$

where p_(E)_ is the prevalence of OSA; HR(t) is hazard ratio at time t.

Finally, when the measure of association provided was odds ratio (OR), PAF was calculated according to the method based on Eide and Heunch (2001) [53] and used in a study by Hillman and colleagues (2018) [43]. By solving simultaneously the following two equations for p_(D|E)_ and p_(D|~E)_

$$p_{\left(D|E\right)}*p_{\left(E\right)}+p_{\left(D|\sim E\right)}*p_{\left(\sim E\right)}=p_{\left(D\right)}$$

$$(\frac{p_{\left(D|E\right)}}{1-p_{\left(D|E\right)}})/(\frac{p_{\left(D|\sim E\right)}}{1-p_{\left(D|\sim E\right)}})=OR$$

the formula for PAF calculation is obtained

$$PAF=\frac{\left(p_{\left(D|E\right)}-p_{\left(D|\sim E\right)}\right)*p_{\left(E\right)}}{p_{\left(D\right)}}$$

where p_(D|E)_ is the probability of having the particular condition given that an individual has OSA; p_(D|~E)_ is the probability of having the particular condition given that an individual does not have OSA; p_(E)_ is the probability of having OSA (i.e., OSA prevalence); p_(~E)_ is the probability of not having OSA; p_(D)_ is the probability of having the particular condition; OR is the odds ratio for that condition for individuals with OSA.

The number of prevalent cases for each condition influenced by OSA and its syndrome was obtained by multiplying conditions’ prevalence by the relative PAF.

### Cost analysis: Assessment of conditions’ costs and estimation of OSA economic burden

An extensive literature review on scientific databases (e.g., ScienceDirect, Scopus) and on Google was carried out in order to retrieve Italian cost data for the conditions included in the systematic review. A top-down approach was used for cost estimation. As a societal perspective was adopted, all cost categories (i.e., direct healthcare, direct non-healthcare and indirect costs) were included, when available. In case we could not retrieve a cost study for Italy, we included cost studies referred to other countries, adjusting for inflation and purchasing power differences. To estimate indirect costs due to all-cause mortality associated with OSA, the friction method was used. This method assumes that for long term absences, as in the case of premature death, an individual’s work can be replaced by the market, therefore the loss in production is limited to a period in which the market adapts to the changed situation, called friction period [54]. This approach is more conservative than the human capital method, which considers earnings lost over a lifetime [55]. In the present analysis, we considered only productivity losses of employed people, excluding indirect costs of people out of the labour market. Productivity costs were estimated for different age groups to account for differences in wages, considering age and gender-specific yearly paid production value [56] and employment rates [48]. People aged 65–74 were assumed to be retired, therefore their employment rate was set equal to 0. To the best of our knowledge, there are not published data on the friction period for Italy, therefore we used a plausible value of 75 days, in line with the estimates used in another European country (Spain) [57]. Moreover, for employed people, we assumed that the friction period was the same across all age groups. The mean cost per patient/year was calculated for each condition and multiplied for the number of prevalent cases influenced by OSA and its syndrome, as obtained by applying PAF methodology.

As OSA is associated with decreased quality of life (QoL) [18–20], we estimated its burden also in terms of quality-adjusted life years (QALYs) value lost due to OSA undertreatment. QALYs for a single patient can be obtained by multiplying the health utility values for the years lived [58]. Health utilities represent individuals’ preferences for different health states and can take on values from 0 (death) to 1 (perfect health) [58]. Since our time perspective is one year, in the present case the QALY for a single patient coincides with the health utility value. In order to express the QALYs lost due to undertreatment in monetary terms, we evaluated them using a willingness-to-pay (WTP) threshold, which represents a measure of the amount of money a society is willing to invest in order to improve health (in this case, to obtain one additional QALY). Health utility and WTP values were retrieved from a review of the literature. In particular, the WTP threshold was sourced from an empirical work carried out by Woods and colleagues [59]. We adopted a conservative approach and considered the lower WTP value reported by the authors (i.e., $16,712, corresponding to €14,860 in 2018). In line with expert opinion, we assumed that only moderate-severe OSA has a substantial impact on patients’ QoL, therefore the present analysis was focused on this patient subpopulation. QALYs value lost was computed according to the following formulas, for alive and dead moderate-severe patients respectively:

$${QALYs\;value\;lost}_{alive}=\left[\left({utility}_{treated}-{utility\;}_{untreated}\right)*\#alive\;untreated\;patients\right]*WTP$$

$${QALYs\;value\;lost}_{dead}=\left[\left({utility\;}_{treated}-\left(\frac{{utility}_{untreated}}{2}+\frac{{utility\;}_{dead}}{2}\right)\right)*\#dead\;untreated\;patients\right]*WTP$$

The total economic burden of OSA for the society was estimated by summing the costs of each condition influenced by the disease, the costs due to OSA’s diagnosis and treatment and the QALYs value lost.

All costs were adjusted for inflation to 2018 (most updated data when analysis was carried out) in their national currency using gross domestic product deflators retrieved from the Organisation for Economic Co-operation and Development (OECD) database [60]. Finally, all costs were adjusted for purchasing power differences using OECD Purchasing Power Parities (PPPs) for GDP for 2018 [60]. PPPs serve both as currency convertors and as spatial price deflators: they convert different currencies to a common currency and, in the process of conversion, equalise their purchasing power by eliminating the differences in price levels between countries. This methodology can ensure better comparability between different currencies. As a one-year time-horizon was considered, no discounting on costs was applied.

### Sensitivity analysis

A one-way deterministic sensitivity analysis was performed in order to account for uncertainty and test robustness of results regarding conditions’ burden influenced by OSA. In particular, all key parameters of the analysis were tested and varied according to 95% confidence intervals (95% CIs) or plausible ranges of variation: OSA prevalence (±10%), conditions’ prevalence (±10%), magnitude of association (95% CIs) and conditions’ costs (±10%). Each variable was tested at the upper and lower limits of its respective interval. Results were graphically reported through a tornado diagram.

## Results

### OSA prevalence in Italy

The review of the literature revealed that epidemiological studies on OSA in Italy are scant [61–63], and the estimates provided were either outdated or focused on a local context, therefore hardly generalizable. Moreover, as OSA is a highly undiagnosed condition, epidemiological studies conducted on sample of individuals with suspected OSA are likely to be biased and capture only diagnosed prevalence, thus underestimating the real one. On the basis of clinical expert opinion, two European-based and one literature-based studies were included for OSA’s prevalence estimate in the Italian population aged 15–74 years. In particular, prevalence data provided by one of the European studies identified (HypnoLaus [31]), a population-based study, were considered both representative of OSA epidemiology in Italy, reflecting the prevalence ratio of 2:1 among men and women observed in the Italian adult population [63], and reliable (i.e., reflecting the actual prevalence), as the sample of individuals tested with polysomnography was randomly drawn from the general population, considering all individuals regardless any OSA suspicions. The literature-based study identified [64], which used publicly available data and expert opinion to estimate the global prevalence of OSA, provided lower prevalence data for Italy than those obtained from the HypnoLaus. Therefore, in order to take into account the full range of variation of prevalence estimates, both studies were included in our analyses. The other European-based study by Hedner and colleagues [65] was used to stratify patients according to OSA severity as measured by AHI. Finally, data on the resident population in Italy in 2018 were sourced from Istat [48] and used to derive the number of prevalent cases. Additional information on prevalence estimates are reported in S1 File.

Results suggest that OSA prevalence in Italy is substantial, with moderate-severe condition (AHI≥15) affecting between 9% and 27% of the population aged 15–74 years (Table 1). On the basis of the data collected by the Italian association of apneic patients (Associazione Apnoici Italiani Onlus), in Italy patients currently treated with continuous and automatic positive airway pressure (the standard of care), are approximately 230,000, representing around 2% (model 1) and 6% (model 2) of the estimated prevalence of moderate-severe OSA. According to expert opinion, the patients currently diagnosed are approximately twofold than those treated (around 4% (model 1) and 12% (model 2) of moderate-severe OSA patients), but still far from the actual prevalence rates. These data suggest a substantial gap in both OSA diagnosis and treatment.

**Table 1:** Prevalence of OSA for the general adult population in Italy (aged 15–74).

	Estimates from the population-based study (model 1)			Estimates from the literature-based study (model 2)		
	Female	Male	Total	Female	Male	Total
***Rates***						
Mild (5≤AHI<15)	29.2%	24.8%	27.1%	7.2%	5.2%	6.2%
Moderate-severe (AHI≥15)	18.3%	36.2%	27.1%	5.9%	11.7%	8.8%
Moderate (15≤AHI<30)	9.8%	14.5%	12.1%	3.2%	4.7%	3.9%
Severe (AHI≥30)	8.5%	21.7%	15.0%	2.8%	7.0%	4.9%
Overall (AHI≥5)	47.5%	61.0%	54.2%	13.1%	16.9%	15.0%
***Absolute values***						
Mild (5≤AHI<15)	6,703,067	5,582,051	12,285,118	1,657,025	1,163,534	2,820,559
Moderate-severe (AHI≥15)	4,193,897	8,135,717	12,329,614	1,354,459	2,627,507	3,981,966
Moderate (15≤AHI<30)	2,236,745	3,260,161	5,496,906	722,378	1,052,900	1,775,278
Severe (AHI≥30)	1,957,152	4,875,556	6,832,708	632,081	1,574,607	2,206,688
Overall (AHI≥5)	10,896,964	13,717,768	24,614,732	3,011,484	3,791,041	6,802,526

Source. Our elaboration from Heinzer et al (2015) [31], Benjafield et al (2019) [64], Hedner et al (2011) [65] and Istat data [48].

### Conditions influenced by OSA

**Fig 1:**
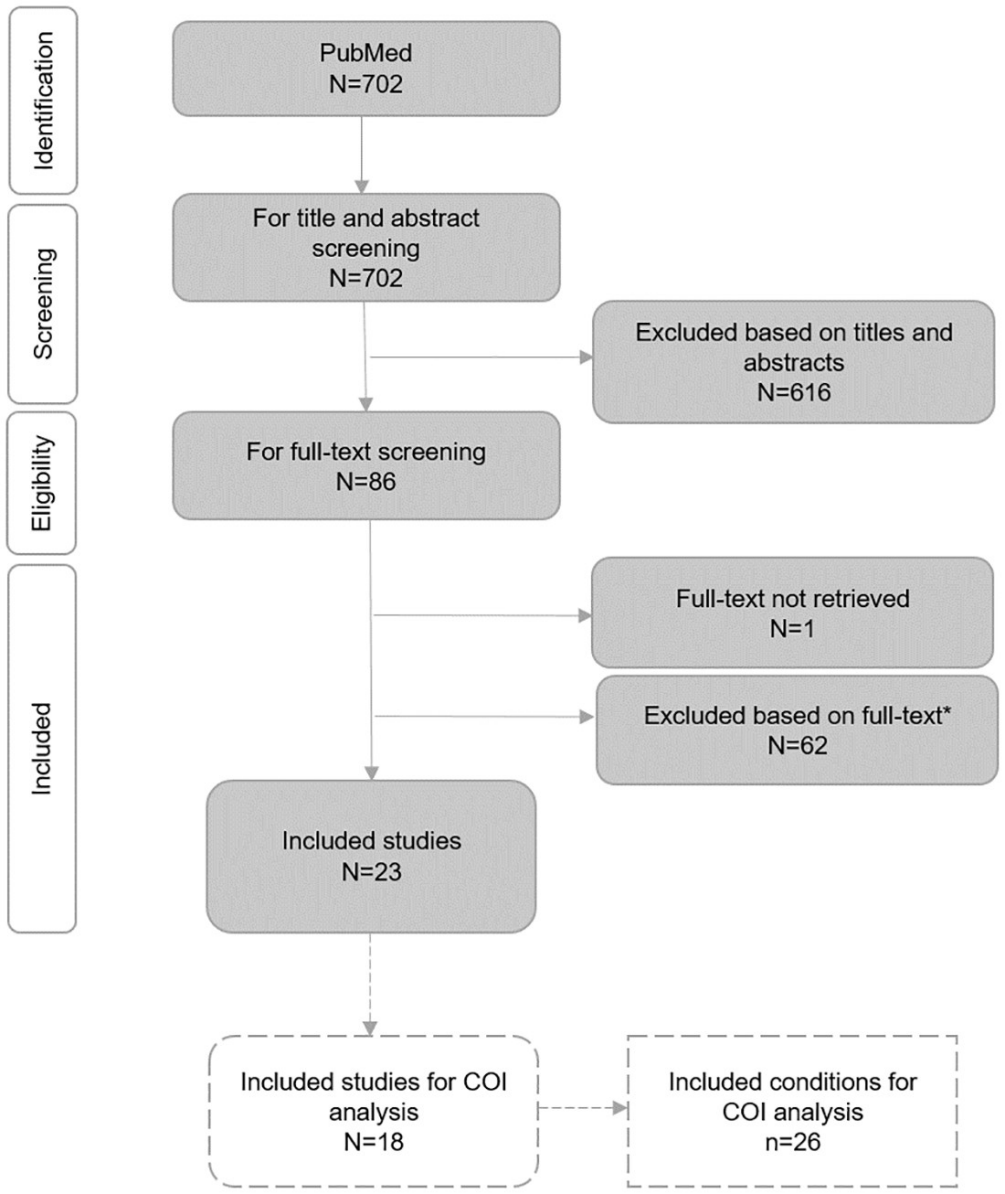
Systematic literature review—Screening process (PRISMA flow diagram). *Reasons for full-text exclusion: 1) OSA exclusively investigated as a consequence of other conditions; 2) no quantitative data provided on the association of OSA with other conditions; 3) focus on parameters that could eventually identify a clinical condition, but not on the clinical condition itself; 4) lack of a control group of non-OSA patients; 5) unclear data on the direction of the association between OSA and the condition investigated; 6) focus on OSA together with other sleep disorders; 7) measure of association that could not be used for PAF calculation; 8) association between increase in AHI and the condition investigated.

Of the 702 studies retrieved, 86 were included for full-text reading (Fig 1), as they did not meet any of the exclusion criteria previously established (S2 Table). If more than one study was available for the same condition, only the most recent and comprehensive one was included. However, if studies on the same condition reported discordant results on the association with OSA, they were all included in the analysis. On the basis of full-texts analysis and reference screening, 23 meta-analyses were selected [66–88] (Fig 1). Unfortunately, for some conditions judged relevant by clinicians involved in the research board (e.g., arrhythmias, psoriasis), screened studies either did not provide sufficient quantitative data on the association or provided an association measure that could not be used for PAF calculation (e.g., Cohen’s d, rate ratio), therefore they were excluded from the analysis. For each condition included, data were extracted according to a predefined template presented in the Methods section (Table 2). Four conditions included in data extraction but either judged not clinically meaningful by experts or for which there is a lack of epidemiological/cost evidence (i.e., spontaneous cerebrospinal fluid leak, floppy eyelids syndrome, nonarteritic anterior ischemic optic neuropathy, pulmonary edema during pregnancy) were ultimately excluded from COI analysis, as well as conditions with a non-statistically significant association (Table 2). Overall, 26 clinical and non-clinical conditions from 18 meta-analyses were considered for the estimation of OSA’s economic burden (Fig 1, Table 2). The PRISMA checklist [46] is provided in the S2 File.

**Table 2:** Results of systematic literature review: Association between OSA and other conditions.

Condition	OSA severity	Association measure	Magnitude	95% CIs	p-value	Included in COI analysis	Source
All-cause mortality	Mild	RR	1.26	0.77–2.07	NS	No	Xie et al (2017) [66]
	Moderate	RR	1.04	0.60–1.79	NS	No	
	Severe	RR	1.54	1.21–1.97	<0.001	Yes	
Cancer mortality	Mild	HR	0.79	0.46–1.34	NS	No	Zhang et al (2017) [67]
	Moderate	HR	1.92	0.63–5.88	NS	No	
	Severe	HR	2.09	0.45–9.81	NS	No	
	Overall	HR	1.38	0.79–2.41	NS	No	
Cardiovascular mortality	Mild	RR	1.80	0.68–4.76	NS	No	Xie et al (2017) [66]
	Moderate	RR	1.11	0.53–2.35	NS	No	
	Severe	RR	2.96	1.45–6.01	0.003	Yes	
Cancer	Mild	HR	0.91	0.74–1.13	NS	No	Zhang et al (2017) [67]
	Moderate	HR	1.07	0.86–1.33	NS	No	
	Severe	HR	1.03	0.85–1.26	NS	No	
	Overall	HR	1.04	0.92–1.16	NS	No	
	Overall	RR	1.40	1.01–1.95	0.04	Yes	Palamaner Subash Shantha et al (2015) [68]
Diabetic retinopathy	Overall	OR	2.01	1.49–2.72	<0.05	Yes	Zhu et al (2017) [69]
Diabetic kidney disease	Overall	OR	1.59	1.16–2.18	<0.05	Yes	Leong et al (2016) [70]
Type 2 diabetes mellitus	Mild	RR	1.22	0.91–1.63	NS	No	Wang et al (2013) [71]
	Moderate-severe	RR	1.63	1.09–2.45	0.018	Yes	
Metabolic syndrome	Mild	OR	2.39	1.65–3.46	<0.05	Yes	Xu et al (2015) [72]
	Moderate-severe	OR	3.42	2.28–5.13	<0.05	Yes	
Depression	Overall	OR^†^	1.12	0.78–1.47	NS	No	Edwards et al (2020) [73]
		RR^‡^	2.18	1.47–2.88	0.005	Yes	
Erectile dysfunction	Overall (men)	RR	1.82	1.12–2.97	<0.05	Yes	Liu et al (2015) [74]
Female sexual dysfunction	Overall (women)	RR	2.00	1.29–3.08	<0.05	Yes	Liu et al (2015) [74]
Parkinson’s disease	Overall	HR	1.59	1.36–1.85	<0.001	Yes	Sun et al (2020) [75]
Stroke	Mild	RR	1.29	0.69–2.41	NS	No	Xie et al (2017) [66]
	Moderate	RR	1.35	0.82–2.23	NS	No	
	Severe	RR	2.15	1.42–3.24	<0.001	Yes	
Spontaneous cerebrospinal fluid leak	Overall	OR	3.43	1.55–7.59	0.002	No	Bakhsheshian et al (2015) [76]
Floppy eyelids syndrome	Overall	OR	4.70	2.98–7.41	<0.001	No	Huon et al (2016) [77]
Glaucoma	Overall	OR	1.24	1.20–1.28	<0.001	Yes	Huon et al (2016) [77]
Nonarteritic anterior ischemic optic neuropathy	Overall	OR	6.18	2.00–19.11	0.002	No	Wu et al (2016) [78]
Resistant hypertension	Overall	OR	2.84	1.70–3.98	<0.05	Yes	Hou et al (2018) [79]
Essential hypertension	Mild	OR	1.18	1.09–1.27	<0.05	Yes	Hou et al (2018) [79]
	Moderate	OR	1.32	1.20–1.43	<0.05	Yes	
	Severe	OR	1.56	1.29–1.84	<0.05	Yes	
Ischemic heart disease	Mild	RR	1.25	0.95–1.66	NS	No	Xie et al (2017) [66]
	Moderate	RR	1.38	1.04–1.83	0.026	Yes	
	Severe	RR	1.63	1.18–2.26	0.003	Yes	
Heart failure	Mild	RR	1.02	0.78–1.34	NS	No	Xie et al (2017) [66]
	Moderate	RR	1.07	0.74–1.54	NS	No	
	Severe	RR	1.44	0.94–2.21	NS	No	
Aortic dissection	Mild	OR	1.60	1.01–2.53	0.04	Yes	Zhou et al (2018) [80]
	Moderate-severe	OR	4.43	2.59–7.59	<0.001	Yes	
Allergic rhinitis	Overall	OR	1.73	0.94–3.20	NS	No	Cao et al (2018) [81]
Non-alcoholic fatty liver disease	Overall	OR	2.34	1.71–3.18	<0.001	Yes	Musso et al (2013) [82]
Gastroesophageal reflux disease	Overall	OR	1.57	1.07–2.08	<0.05	Yes	Wu et al (2018) [83]
Gout	Overall	HR	1.25	0.91–1.70	NS	No	Shi et al (2019) [84]
Pre-eclampsia	Overall (women)	OR	2.35	2.15–2.58	<0.001	Yes	Liu et al (2019) [85]
Gestational hypertension	Overall (women)	OR	1.97	1.51–2.56	<0.001	Yes	Liu et al (2019) [85]
Gestational diabetes	Overall (women)	RR	1.40	0.62–3.19	NS	No	Xu et al (2014) [86]
	Overall (women)	OR	1.55	1.26–1.90	<0.001	Yes	Liu et al (2019) [85]
Preterm delivery	Overall (women)	OR	1.62	1.29–2.02	<0.001	Yes	Liu et al (2019) [85]
Cesarean delivery	Overall (women)	OR	1.42	1.12–1.79	<0.001	Yes	Liu et al (2019) [85]
Pulmonary edema	Overall (women)*	OR	6.35	4.25–9.50	<0.001	No	Liu et al (2019) [85]
Car accidents	Overall	OR	2.43	1.21–4.89	0.013	Yes	Tregear et al (2009) [87]
Work accidents	Overall	OR	1.78	1.03–3.07	<0.001	Yes	Garbarino et al (2016) [88]

Note.

^†^Estimates obtained from a meta-analysis of cross-sectional studies.

^‡^Estimates were obtained from a meta-analysis of longitudinal studies.

*The focus was only on pregnant women.

### Conditions’ burden associated with OSA

For the conditions included, epidemiological data were retrieved from different sources [48, 89–110] and reported in S3 File. Conditions’ prevalence data (or incidence when appropriate) were used, together with data on magnitude of association (Table 2) and prevalence of OSA (Table 1), to estimate the proportion of burden associated with OSA through PAF methodology [43, 51, 52]. The PAF for car and work accidents was derived considering only OSA population with excessive daytime sleepiness (EDS), estimated at 19% [35]. Moreover, for the conditions providing only estimates for overall OSA, a conservative approach was adopted and PAF was calculated considering moderate-severe subpopulation as, according to expert opinion, these patients are more likely to develop comorbidities than mild ones. Table 3 provides the results for PAF (i.e., the proportion of each condition influenced by the presence of OSA and its syndrome) and the total number of cases/year for each condition, calculated using OSA prevalence data from either the population-based (model 1) or the literature-based study (model 2), and stratified by OSA severity.

**Table 3:** Conditions’ burden influenced by OSA: PAF and number of cases/year among general adult population in Italy (aged 15–74 years).

Condition	OSA severity	Model 1		Model 2		Source of epidemiological data
		PAF	Number of cases/year	PAF	Number of cases/year	
All-cause mortality	Severe	7.5%	11,129	2.6%	3,797	Istat [48]
Cardiovascular mortality	Severe	22.7%	7,377	8.7%	2,823	Istat [48]
Cancer	Overall*	9.7%	184,224	3.4%	64,038	AIOM-AIRTUM (2018) [89]
Diabetic retinopathy	Overall*	20.7%	246,930	7.8%	92,650	AMD et al (2015) [90]
Diabetic kidney disease	Overall*	13.5%	92,915	4.8%	33,131	AMD-SID (2018) [91]
						IDF (2017) [92]
Type 2 diabetes	Moderate-severe	14.5%	450,426	5.2%	162,188	IDF (2017) [92]
Metabolic syndrome	Mild	16.4%	2,454,641	3.9%	587,706	Tocci et al (2015) [93]
	Moderate-severe	23.2%	3,470,981	7.8%	1,171,728	
Depression^◻^	Overall*	24.2%	175,121	9.4%	67,954	Istat (2018) [94]
Erectile dysfunction	Overall (men)*	22.8%	511,257	8.7%	196,081	Nicolosi et al (2003) [95]
Female sexual dysfunction	Overall (women)*	15.3%	1,014,992	5.6%	371,226	Graziottin (2007) [96]
Parkinson’s disease	Overall*	13.7%	7,726	4.9%	2,766	Riccò et al (2020) [97]
Stroke†	Severe	14.7%	10,757	5.3%	3,869	Stevens et al (2017) [98]
Glaucoma	Overall*	6.0%	48,430	2.0%	16,373	Tham et al (2014) [99]
Resistant hypertension	Overall*	32.5%	235,129	13.4%	97,135	Giampaoli et al (2015) [100]
						Dovellini (2000) [101]
Essential hypertension	Mild	3.2%	442,561	0.8%	103,155	Giampaoli et al (2015) [100]
	Moderate	2.4%	327,235	0.8%	107,445	
	Severe	4.9%	673,131	1.6%	221,359	Dovellini (2000) [101]
Ischemic heart disease	Moderate	4.4%	99,296	1.5%	33,325	Giampaoli et al (2015) [100]
	Severe	8.6%	196,584	3.0%	67,625	
Aortic dissection†	Mild	13.9%	224	3.6%	58	Pacini et al (2013) [102]
	Moderate-severe	48.1%	774	23.1%	372	
Non-alcoholic fatty liver disease	Overall*	19.9%	1,844,121	7.0%	653,912	Younossi et al (2016) [103]
Gastroesophageal reflux disease	Overall*	11.0%	536,097	3.8%	185,754	Darbà et al (2011) [104]
Pre-eclampsia	Overall (women)*	19.5%	1,790	7.4%	676	Fox et al (2017) [105]
Gestational hypertension	Overall (women)*	14.9%	2,041	5.4%	744	FIGO (2016) [106]
Gestational diabetes	Overall (women)*	9.0%	4,486	3.1%	1,567	Meregaglia et al (2018) [107]
Preterm delivery	Overall (women)*	10.0%	2,801	3.5%	986	Merinopoulou et al (2018) [108]
Cesarean delivery	Overall (women)*	7.0%	11,525	2.4%	3,967	OECD [109]
Car accidents ‡	Overall*	5.3%	11,420	2.1%	92,650	Istat-Aci (2017) [110]
Work accidents ‡	Overall*	3.3%	845	1.2%	33,131	Istat-Aci (2017) [110]

Note. Model 1: Statistics calculated using OSA prevalence data derived from the population-based study. Model 2: Statistics calculated using OSA prevalence data derived from the literature-based study.

^◻^Estimates are referred to major depression.

^†^Incidence data were considered.

^‡^Only OSA population with excessive daytime sleepiness was considered for PAF calculation.

*Only conservative estimates (i.e., referred to moderate-severe subpopulation) were provided.

### OSA economic burden

For all-cause and cardiovascular premature mortality, indirect costs were estimated using the friction cost method, for both model 1 and model 2 (S4 File). Cost data of all other conditions were retrieved from the literature [107, 111–129] and expressed in 2018 Euros standardized for inflation and PPP (S5 File). In order to avoid double counting, only productivity losses due to morbidity were considered for these conditions when the original study reported separate estimates for costs due morbidity and mortality, as indirect costs due to mortality were computed separately. Unfortunately, for some conditions, it was not possible to retrieve an Italian cost study. Moreover, the majority of studies did not report estimates for all cost categories, leading to a possible underestimation of the overall economic burden. Through the multiplication of the cost per patient/year by the number of prevalent (or incident) cases due to OSA, we estimated the total economic burden influenced by OSA in Italy in one year. Mean estimates are provided in Table 4. Results suggest that the economic burden due to conditions influenced by OSA in Italy is substantial and ranges from €10.7 billion (model 2) to €32.0 billion (model 1) per year, corresponding to €177 and €530 per Italian resident respectively. The main driver of economic burden are direct healthcare costs, which account for 60% and 57% of total cost according to model 1 and model 2 respectively, followed by indirect costs (37% and 39%) and direct non-healthcare costs (both 4%). Considering the health expenditure per capita in Italy in 2018 (€3,429) [130], the direct healthcare costs per resident generated by conditions influenced by OSA represent between the 3% (model 2) and 9% (model 1) of national health expenditure.

**Table 4:** Annual economic burden of conditions influenced by OSA in Italy.

	Model 1				Model 2			
Condition	Direct healthcare cost	Direct non-healthcare cost	Productivity losses cost*	Total cost	Direct healthcare cost	Direct non-healthcare cost	Productivity losses cost*	Total cost
Mortality †			€ 17,468,314	€ 17,468,314			€ 5,960,283	€ 5,960,283
Cancer	€ 1,053,335,086	€ 843,866,787	€ 21,976,498	€ 1,919,178,370	€ 366,149,501	€ 293,336,287	€ 7,639,244	€ 667,125,033
Diabetic retinopathy	€ 75,903,881	€ 59,787,476	€ 142,872,419	€ 278,563,776	€ 28,479,821	€ 22,432,800	€ 53,607,020	€ 104,519,641
Diabetic kidney disease	€ 74,076,551			€ 74,076,551	€ 26,413,493			€ 26,413,493
Type 2 diabetes	€ 1,741,141,727		€ 1,960,219,449	€ 3,701,361,176	€ 626,945,056		€ 705,829,901	€ 1,332,774,957
Metabolic syndrome	€ 11,260,422,980		€ 531,818,651	€ 11,792,241,631	€ 3,343,442,091		€ 157,907,466	€ 3,501,349,558
Depression^◻^	€ 152,472,494	€ 86,853,842	€ 340,910,896	€ 580,237,232	€ 59,165,781	€ 33,702,967	€ 132,287,858	€ 225,156,606
Erectile dysfunction	€ 208,151,669			€ 208,151,669	€ 79,831,780			€ 79,831,780
Female sexual dysfunction	€ 772,808,563			€ 772,808,563	€ 282,649,080			€ 282,649,080
Parkinson’s disease	€ 47,486,623	€ 37,281,979	€ 9,360,588	€ 94,129,190	€ 16,997,509	€ 13,344,827	€ 3,350,558	€ 33,692,894
Stroke	€ 144,697,413	€ 91,324,306	€ 9,755,712	€ 245,777,431	€ 52,047,358	€ 32,849,162	€ 3,509,109	€ 88,405,629
Glaucoma	€ 47,690,291			€ 47,690,291	€ 16,122,559			€ 16,122,559
Resistant hypertension	€ 56,172,997			€ 56,172,997	€ 23,205,874			€ 23,205,874
Essential hypertension	€ 344,718,771			€ 344,718,771	€ 103,196,342			€ 103,196,342
Ischemic heart disease	€ 442,622,880	€ 103,029,886	€ 135,642,496	€ 681,295,262	€ 151,016,288	€ 35,152,252	€ 46,279,185	€ 232,447,725
Aortic dissection	€ 37,984,396			€ 37,984,396	€ 16,358,614			€ 16,358,614
Non-alcoholic fatty liver disease	€ 2,208,249,940		€ 8,158,942,384	€ 10,367,192,324	€ 783,029,477		€ 2,893,102,030	€ 3,676,131,507
Gastroesophageal reflux disease	€ 165,097,914		€ 99,881,300	€ 264,979,215	€ 57,205,202		€ 34,608,129	€ 91,813,331
Pre-eclampsia	€ 8,356,628			€ 8,356,628	€ 3,157,267			€ 3,157,267
Gestational hypertension	€ 24,202,545			€ 24,202,545	€ 8,824,400			€ 8,824,400
Gestational diabetes	€ 16,844,415			€ 16,844,415	€ 5,883,403			€ 5,883,403
Preterm delivery	€ 25,281,917		€ 27,402,594	€ 52,684,511	€ 8,897,225		€ 9,643,535	€ 18,540,759
Cesarean delivery	€ 28,991,021		€ 10,865,180	€ 39,856,201	€ 9,979,392		€ 3,740,051	€ 13,719,443
Car accidents	€ 106,754,952		€ 272,184,561	€ 378,939,513	€ 42,564,671		€ 108,523,736	€ 151,088,407
Work accidents	€ 7,900,413		€ 20,143,051	€ 28,043,464	€ 2,906,425		€ 7,410,278	€ 10,316,703
**Total**	**€ 19,051,366,068**	**€ 1,222,144,276**	**€ 11,759,444,093**	**€ 32,032,954,437**	**€ 6,114,468,608**	**€ 430,818,296**	**€ 4,173,398,383**	**€ 10,718,685,287**

Note. Model 1: Statistics calculated using OSA prevalence data derived from the population-based study. Model 2: Statistics calculated using OSA prevalence data derived from the literature-based study.

^†^Costs due to cardiovascular mortality were included in all-cause mortality costs in order to avoid double counting.

^◻^Estimates are referred to major depression.

*Only productivity losses due to morbidity were included for conditions different from mortality when the original study reported separate estimates for costs due morbidity and mortality.

The yearly per patient cost of OSA diagnosis and treatment in Italy amounts to approximately €381 and €256, respectively (see S6 File for all details on data sources and calculation). According to the Italian association of apneic patients (Associazione Apnoici Italiani Onlus) and expert opinion, in Italy there are approximately 460,000 patients diagnosed and 230,000 treated. Therefore, the total yearly cost of OSA diagnosis and treatment amounts to €175,347,041 and €58,880,000 respectively, with an overall cost of €234,227,041.

As regards estimation of QALYs value lost due to undertreatment, health utility values were derived from a study by Català and colleagues [131], who found a significant difference in utility values between treated (utility = 0.84) and untreated patients (utility = 0.79). By using these values in the formulas presented in the methods section, we estimated a QALYs value lost ranging from €2.8 billion (model 2) to €9.0 billion (model 1) (see S7 File for additional details on calculation).

As summarized by Table 5, in Italy the total economic burden of OSA ranges from around € 13.8 billion/year (model 2) to €41.3 billion/year (model 2).

**Table 5:** Total annual economic burden of OSA in Italy.

	Model 1	Model 2
OSA diagnosis and treatment	€ 234,227,041	€ 234,227,041
Conditions influenced by OSA	€ 32,032,954,437	€ 10,718,685,287
QALYs value lost	€ 9,029,365,722	€ 2,801,136,971
**Total economic burden**	**€ 41,296,547,200**	**€ 13,754,049,299**

### Sensitivity analysis

**Fig 2:**
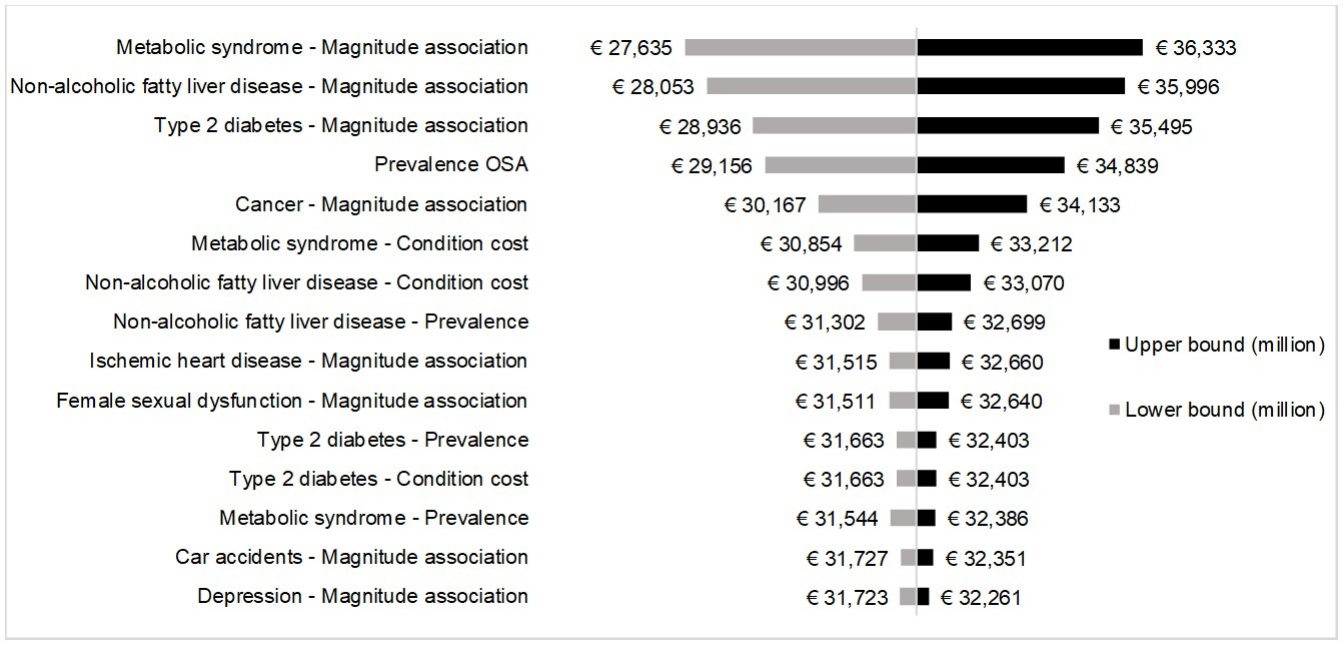
Tornado plot for sensitivity analysis (model 1).

**Fig 3:**
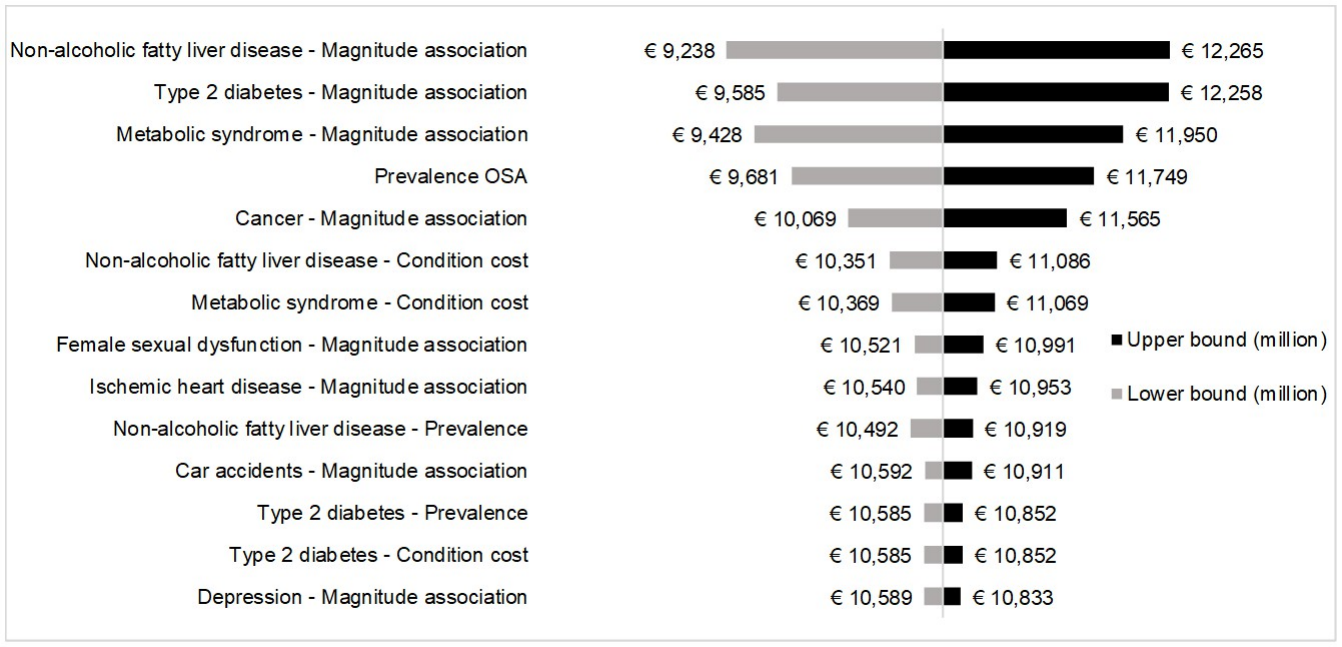
Tornado plot for sensitivity analysis (model 2).

All key parameters used to estimate conditions’ burden influenced by OSA were tested in one-way deterministic sensitivity analysis. The majority of them, however, did not significantly influence the results obtained in the base-case analysis. The tornado plot shows only those variables whose variation caused at least 1% increase or decrease of base-case result. For both model 1 (Fig 2) and model 2 (Fig 3), the five parameters with the highest impact on conditions’ burden influenced by OSA were the magnitude of association with OSA of metabolic syndrome, non-alcoholic fatty liver disease, type 2 diabetes and cancer, and OSA prevalence.

## Discussion

This study aimed at providing reliable estimates of the extent of OSA consequences in Italy and assessing the societal economic burden associated with OSA in the adult population by performing a COI analysis. Several studies demonstrated that OSA is a severely underdiagnosed condition worldwide [1, 132, 133]. We estimated a prevalence of moderate-severe OSA in Italy ranging from approximately 4 to 12 million patients (9% to 27% of the adult population). However, the number of diagnosed and treated patients is substantially lower (around 460,000 and 230,000 patients respectively), suggesting a huge gap in both OSA diagnosis and treatment. The reasons underlying poor diagnosis of OSA are several, and start from lack of awareness [134, 135], both among healthcare professionals and general population, to limited routine screening and diagnostic sleep centres [136]. Even when a diagnosis occurred, evidence shows that acceptance and adherence to treatment with CPAP—despite its technological advances—is generally low, ranging from 30 to 60% [137, 138].

The systematic literature review revealed that several clinical and non-clinical conditions were found to be significantly influenced by OSA and its syndrome. Through the PAF approach, we attributed a portion of each conditions’ burden to OSA, which allowed us to estimate the economic impact associated with the sleep disorder. Results of COI revealed that the economic burden for the society due to conditions associated with OSA in Italy is very high, ranging from €10.7 to €32.0 billion per year, with the main cost driver represented by direct healthcare costs. Moreover, the QALYs value lost due to OSA undertreatment is substantial, and contributes at increasing OSA’s yearly economic burden by €2.8 to €9.0 billion.

Several studies demonstrated that appropriate diagnostic and therapeutic pathways for OSA may have a substantial impact in reducing clinical and non-clinical consequences related to the disease, whereas untreated disease may result in increased clinical and economic burden [3, 139]. In particular, therapy with CPAP was demonstrated to be effective in preventing the onset and reducing the burden of some of the associated conditions, including all-cause and cardiovascular mortality [140], stroke [141], car [142] and work accidents [143]. Overall, these results suggest that OSA’s underdiagnosis and undertreatment have a detrimental effect both on the onset of associated conditions and on patients’ quality of life, ultimately leading to loss of value for the society. An increasing awareness towards the disease is fundamental in order to implement appropriate diagnostic and treatment pathways for OSA patients and reduce its substantial clinical and economic burden.

### Study limitations

This study has some limitations. First, the review performed may not be fully exhaustive as we did not consider individual studies but focused only on systematic reviews and meta-analyses, including the latter for COI analysis. The reason underlying this choice is that evidence on OSA’s association with other conditions is very heterogeneous, therefore we opted for the “systematic review of systematic reviews” method [47], which is recommended in all cases where individual studies are heterogeneous in terms of methods, definitions and results. Moreover, we further restricted the selection on systematic reviews presenting a meta-analysis because we needed a reliable quantitative assessment of the intensity of association between OSA and other conditions. Although a vast literature exists on OSA association with other conditions, in some cases single studies either provide insufficient quantitative evidence or no quantitative evidence at all. Second, due to lack of Italian data, we had to rely on OSA epidemiological estimates derived from two different studies, a population-based study conducted in another European country and a model-based study. Estimated prevalence data, although validated by a clinical expert, should be interpreted cautiously. Further research is needed in order to provide up-to-date evidence on OSA epidemiology in Italy, which in turn might increase awareness of the extent of conditions’ burden for our country. Third, few conditions’ prevalence data were lacking for Italy, and we had to use estimates derived from other countries. The same happened for cost data, although we adjusted for purchasing power differences to ensure better comparability between different currencies. Moreover, it was not always possible to retrieve data on all relevant cost components, namely direct non-healthcare costs and productivity losses due to morbidity, potentially leading to an underestimation of OSA economic burden.

## Conclusions

Results of the present COI analysis suggest that the burden of OSA in Italy is substantial but subtle, because it is greatly hidden behind the cost of other conditions for which OSA is a risk factor. Moreover, underdiagnosis and low treatment rates are observed. More appropriate diagnosis rates and clinical pathways for OSA patients, in particular for moderate-severe population, are recommended.

## Supporting information

S1 Table Search strategy. (DOCX)

S2 Table Exclusion criteria. (DOCX)

S1 File Additional information on OSA prevalence estimation. (DOCX)

S2 File PRISMA checklist. (DOCX)

S3 File Prevalence of conditions significantly associated with OSA. (DOCX)

S4 File Indirect costs due to all-cause and cardiovascular mortality. (DOCX)

S5 File Cost of conditions. (DOCX)

S6 File Cost of OSA diagnosis and treatment. (DOCX)

S7 File Additional information on QALYs value lost calculation. (DOCX)

## References

[1] Young T, Palta M, Dempsey J, Skatrud J, Weber S, Badr S. The occurrence of sleep-disordered breathing among middle-aged adults. N Engl J Med. 1993;328(17):1230–5. doi: 10.1056/NEJM199304293281704 8464434

[2] McNicholas WT, Bonsigore MR, Management Committee of of EU COST ACTION B26. Sleep apnoea as an independent risk factor for cardiovascular disease: current evidence, basic mechanisms and research priorities. Eur Respir J. 2007;29(1):156–78. doi: 10.1183/09031936.00027406 17197482

[3] Knauert M, Naik S, Gillespie MB, Kryger M. Clinical consequences and economic costs of untreated obstructive sleep apnea syndrome. World Journal of Otorhinolaryngology—Head and Neck Surgery. 2015;1(1):17–27. doi: 10.1016/j.wjorl.2015.08.001 29204536 PMC5698527

[4] Malhotra A, White DP. Obstructive sleep apnoea. Lancet. 2002;360(9328):237–45. doi: 10.1016/S0140-6736(02)09464-3 12133673

[5] McNicholas WT. Diagnosis of obstructive sleep apnea in adults. Proc Am Thorac Soc. 2008;5(2):154–60. doi: 10.1513/pats.200708-118MG 18250207

[6] Park JG, Ramar K, Olson EJ. Updates on definition, consequences, and management of obstructive sleep apnea. Mayo Clin Proc. 2011;86(6):549–54; quiz 54–5. doi: 10.4065/mcp.2010.0810 21628617 PMC3104914

[7] Punjabi NM. The epidemiology of adult obstructive sleep apnea. Proc Am Thorac Soc. 2008;5(2):136–43. doi: 10.1513/pats.200709-155MG 18250205 PMC2645248

[8] Kent BD, Grote L, Ryan S, Pepin JL, Bonsignore MR, Tkacova R, et al. Diabetes mellitus prevalence and control in sleep-disordered breathing: the European Sleep Apnea Cohort (ESADA) study. Chest. 2014;146(4):982–90. doi: 10.1378/chest.13-2403 24831859

[9] Reichmuth KJ, Austin D, Skatrud JB, Young T. Association of sleep apnea and type II diabetes: a population-based study. Am J Respir Crit Care Med. 2005;172(12):1590–5. doi: 10.1164/rccm.200504-637OC 16192452 PMC2718458

[10] Ronksley PE, Hemmelgarn BR, Heitman SJ, Hanly PJ, Faris PD, Quan H, et al. Obstructive sleep apnoea is associated with diabetes in sleepy subjects. Thorax. 2009;64(10):834–9. doi: 10.1136/thx.2009.115105 19679579

[11] Cao J, Feng J, Li L, Chen B. Obstructive sleep apnea promotes cancer development and progression: a concise review. Sleep Breath. 2015;19(2):453–7. doi: 10.1007/s11325-015-1126-x 25643765

[12] Gozal D, Ham SA, Mokhlesi B. Sleep Apnea and Cancer: Analysis of a Nationwide Population Sample. Sleep. 2016;39(8):1493–500. doi: 10.5665/sleep.6004 27166241 PMC4945307

[13] Shahar E, Whitney CW, Redline S, Lee ET, Newman AB, Nieto FJ, et al. Sleep-disordered breathing and cardiovascular disease: cross-sectional results of the Sleep Heart Health Study. Am J Respir Crit Care Med. 2001;163(1):19–25. doi: 10.1164/ajrccm.163.1.2001008 11208620

[14] Marin JM, Carrizo SJ, Vicente E, Agusti AG. Long-term cardiovascular outcomes in men with obstructive sleep apnoea-hypopnoea with or without treatment with continuous positive airway pressure: an observational study. Lancet. 2005;365(9464):1046–53. doi: 10.1016/S0140-6736(05)71141-7 15781100

[15] Arzt M, Young T, Finn L, Skatrud JB, Bradley TD. Association of sleep-disordered breathing and the occurrence of stroke. Am J Respir Crit Care Med. 2005;172(11):1447–51. doi: 10.1164/rccm.200505-702OC 16141444 PMC2718439

[16] Yaggi HK, Concato J, Kernan WN, Lichtman JH, Brass LM, Mohsenin V. Obstructive sleep apnea as a risk factor for stroke and death. N Engl J Med. 2005;353(19):2034–41. doi: 10.1056/NEJMoa043104 16282178

[17] Redline S, Yenokyan G, Gottlieb DJ, Shahar E, O’Connor GT, Resnick HE, et al. Obstructive sleep apnea-hypopnea and incident stroke: the sleep heart health study. Am J Respir Crit Care Med. 2010;182(2):269–77. doi: 10.1164/rccm.200911-1746OC 20339144 PMC2913239

[18] Gall R, Isaac L, Kryger M. Quality of life in mild obstructive sleep apnea. Sleep. 1993;16(8 Suppl):S59–61. doi: 10.1093/sleep/16.suppl_8.s59 8178027

[19] Smith IE, Shneerson JM. Is the SF 36 sensitive to sleep disruption? A study in subjects with sleep apnoea. J Sleep Res. 1995;4(3):183–8. doi: 10.1111/j.1365-2869.1995.tb00167.x 10607157

[20] Parish JM, Lyng PJ. Quality of life in bed partners of patients with obstructive sleep apnea or hypopnea after treatment with continuous positive airway pressure. Chest. 2003;124(3):942–7. doi: 10.1378/chest.124.3.942 12970021

[21] AlGhanim N, Comondore VR, Fleetham J, Marra CA, Ayas NT. The economic impact of obstructive sleep apnea. Lung 2008. p. 7–12. doi: 10.1007/s00408-007-9055-5 18066623

[22] Sherman B. Obstructive sleep apnea and health benefits purchasing: an employer perspective. J Clin Sleep Med. 2013;9(3):187–9. doi: 10.5664/jcsm.2472 23495337 PMC3578687

[23] Tarasiuk A, Reuveni H. The economic impact of obstructive sleep apnea. Curr Opin Pulm Med. 2013;19(6):639–44. doi: 10.1097/MCP.0b013e3283659e1e 24060978

[24] Ellen RL, Marshall SC, Palayew M, Molnar FJ, Wilson KG, Man-Son-Hing M. Systematic review of motor vehicle crash risk in persons with sleep apnea. J Clin Sleep Med. 2006;2(2):193–200. 17557495

[25] Karimi M, Hedner J, Habel H, Nerman O, Grote L. Sleep apnea-related risk of motor vehicle accidents is reduced by continuous positive airway pressure: Swedish Traffic Accident Registry data. Sleep. 2015;38(3):341–9. doi: 10.5665/sleep.4486 25325460 PMC4335527

[26] Bearpark H, Elliott L, Grunstein R, Cullen S, Schneider H, Althaus W, et al. Snoring and sleep apnea. A population study in Australian men. Am J Respir Crit Care Med. 1995;151(5):1459–65. doi: 10.1164/ajrccm.151.5.7735600 7735600

[27] Bixler EO, Vgontzas AN, Ten Have T, Tyson K, Kales A. Effects of age on sleep apnea in men: I. Prevalence and severity. Am J Respir Crit Care Med. 1998;157(1):144–8. doi: 10.1164/ajrccm.157.1.9706079 9445292

[28] Bixler EO, Vgontzas AN, Lin HM, Ten Have T, Rein J, Vela-Bueno A, et al. Prevalence of sleep-disordered breathing in women: effects of gender. Am J Respir Crit Care Med. 2001;163(3 Pt 1):608–13. doi: 10.1164/ajrccm.163.3.9911064 11254512

[29] Duran J, Esnaola S, Rubio R, Iztueta A. Obstructive sleep apnea-hypopnea and related clinical features in a population-based sample of subjects aged 30 to 70 yr. Am J Respir Crit Care Med. 2001;163(3 Pt 1):685–9. doi: 10.1164/ajrccm.163.3.2005065 11254524

[30] Young T, Peppard PE, Gottlieb DJ. Epidemiology of obstructive sleep apnea: a population health perspective. Am J Respir Crit Care Med. 2002;165(9):1217–39. doi: 10.1164/rccm.2109080 11991871

[31] Heinzer R, Vat S, Marques-Vidal P, Marti-Soler H, Andries D, Tobback N, et al. Prevalence of sleep-disordered breathing in the general population: the HypnoLaus study. Lancet Respir Med. 2015;3(4):310–8. doi: 10.1016/S2213-2600(15)00043-0 25682233 PMC4404207

[32] Lee W, Nagubadi S, Kryger MH, Mokhlesi B. Epidemiology of Obstructive Sleep Apnea: a Population-based Perspective. Expert Rev Respir Med. 2008;2(3):349–64. doi: 10.1586/17476348.2.3.349 19690624 PMC2727690

[33] Lindberg E, Gislason T. Epidemiology of sleep-related obstructive breathing. Sleep Med Rev. 2000;4(5):411–33. doi: 10.1053/smrv.2000.0118 17210275

[34] Reuveni H, Tarasiuk A, Wainstock T, Ziv A, Elhayany A, Tal A. Awareness level of obstructive sleep apnea syndrome during routine unstructured interviews of a standardized patient by primary care physicians. Sleep. 2004;27(8):1518–25. doi: 10.1093/sleep/27.8.1518 15683143

[35] Young T, Evans L, Finn L, Palta M. Estimation of the clinically diagnosed proportion of sleep apnea syndrome in middle-aged men and women. Sleep. 1997;20(9):705–6. doi: 10.1093/sleep/20.9.705 9406321

[36] Young T, Palta M, Dempsey J, Peppard PE, Nieto FJ, Hla KM. Burden of sleep apnea: rationale, design, and major findings of the Wisconsin Sleep Cohort study. WMJ. 2009;108(5):246–9. 19743755 PMC2858234

[37] Ronksley PE, Hemmelgarn BR, Tsai WH. Comorbidity and healthcare costs in patients with obstructive sleep apnoea. Breathe. 2011;8(2):95–104. doi: 10.1183/20734735.011311

[38] McMillan A, Bratton DJ, Faria R, Laskawiec-Szkonter M, Griffin S, Davies RJ, et al. A multicentre randomised controlled trial and economic evaluation of continuous positive airway pressure for the treatment of obstructive sleep apnoea syndrome in older people: PREDICT. Health Technol Assess. 2015;19(40):1–188. doi: 10.3310/hta19400 26063688 PMC4780948

[39] Jenkinson C, Davies RJ, Mullins R, Stradling JR. Comparison of therapeutic and subtherapeutic nasal continuous positive airway pressure for obstructive sleep apnoea: a randomised prospective parallel trial. Lancet. 1999;353(9170):2100–5. doi: 10.1016/S0140-6736(98)10532-9 10382693

[40] Siccoli MM, Pepperell JC, Kohler M, Craig SE, Davies RJ, Stradling JR. Effects of continuous positive airway pressure on quality of life in patients with moderate to severe obstructive sleep apnea: data from a randomized controlled trial. Sleep. 2008;31(11):1551–8. doi: 10.1093/sleep/31.11.1551 19014075 PMC2579969

[41] Pepperell JC, Ramdassingh-Dow S, Crosthwaite N, Mullins R, Jenkinson C, Stradling JR, et al. Ambulatory blood pressure after therapeutic and subtherapeutic nasal continuous positive airway pressure for obstructive sleep apnoea: a randomised parallel trial. Lancet. 2002;359(9302):204–10. doi: 10.1016/S0140-6736(02)07445-7 11812555

[42] Chen X, Niu X, Xiao Y, Dong J, Lu M, Kong W. Effect of continuous positive airway pressure on leptin levels in patients with obstructive sleep apnea: a meta-analysis. Otolaryngol Head Neck Surg. 2015;152(4):610–8. doi: 10.1177/0194599814562719 25527507

[43] Hillman D, Mitchell S, Streatfeild J, Burns C, Bruck D, Pezzullo L. The economic cost of inadequate sleep. Sleep. 2018;41(8). doi: 10.1093/sleep/zsy083 29868785

[44] Frost & Sullivan. Hidden Health Crisis Costing America Billions. Underdiagnosing and Undertreating Obstructive Sleep Apnea Draining Healthcare System. Darien, IL: American Academy of Sleep Medicine; 2016.

[45] Harvard Medical School & McKinsey Company. The price of fatigue: The surprising economic costs of unmanaged sleep apnea. 2010.

[46] Moher D, Liberati A, Tetzlaff J, Altman DG, Group P. Preferred reporting items for systematic reviews and meta-analyses: the PRISMA statement. PLoS Med. 2009;6(7):e1000097. doi: 10.1371/journal.pmed.1000097 19621072 PMC2707599

[47] Smith V, Devane D, Begley CM, Clarke M. Methodology in conducting a systematic review of systematic reviews of healthcare interventions. BMC Med Res Methodol. 2011;11(1):15. doi: 10.1186/1471-2288-11-15 21291558 PMC3039637

[48] Istituto nazionale di statistica (Istat). Statistiche Istat [Last access: 9th April 2019]. http://dati.istat.it/.

[49] Rockhill B, Newman B, Weinberg C. Use and misuse of population attributable fractions. Am J Public Health. 1998;88(1):15–9. doi: 10.2105/ajph.88.1.15 9584027 PMC1508384

[50] Rosen L. An intuitive approach to understanding the attributable fraction of disease due to a risk factor: the case of smoking. Int J Environ Res Public Health. 2013;10(7):2932–43. doi: 10.3390/ijerph10072932 23863613 PMC3734469

[51] Levin ML. The occurrence of lung cancer in man. Acta Unio Int Contra Cancrum. 1953;9(3):531–41. 13124110

[52] Samuelsen SO, Eide GE. Attributable fractions with survival data. Stat Med. 2008;27(9):1447–67. doi: 10.1002/sim.3022 17694507

[53] Eide GE, Heuch I. Attributable fractions: fundamental concepts and their visualization. Stat Methods Med Res. 2001;10(3):159–93. doi: 10.1177/096228020101000302 11446147

[54] Koopmanschap MA, van Ineveld BM. Towards a new approach for estimating indirect costs of disease. Soc Sci Med. 1992;34(9):1005–10. doi: 10.1016/0277-9536(92)90131-9 1631600

[55] Mushkin SJ. Health as an Investment. Journal of Political Economy. 1962;70(5, Part 2):129–57. doi: 10.1086/258730

[56] Pradelli L, Ghetti G. A general model for the estimation of societal costs of lost production and informal care in Italy. Farmeconomia Health economics and therapeutic pathways; Vol 18, No 1 (2017)DO—doi: 10.7175/fe.v18i11.278 2017.

[57] Oliva J, Lobo F, Lopez-Bastida J, Zozaya N, Romay R. Indirect costs of cervical and breast cancers in Spain. Eur J Health Econ. 2005;6(4):309–13. doi: 10.1007/s10198-005-0303-4 16133097

[58] Weinstein MC, Torrance G, McGuire A. QALYs: the basics. Value Health. 2009;12 Suppl 1:S5–9. doi: 10.1111/j.1524-4733.2009.00515.x 19250132

[59] Woods B, Revill P, Sculpher M, Claxton K. Country-Level Cost-Effectiveness Thresholds: Initial Estimates and the Need for Further Research. Value Health. 2016;19(8):929–35. doi: 10.1016/j.jval.2016.02.017 27987642 PMC5193154

[60] OECD. OECD.Stat—GDP deflators, forecast growth [Last access: 9th April 2019]. https://stats.oecd.org.

[61] Cirignotta F, D’Alessandro R, Partinen M, Zucconi M, Cristina E, Gerardi R, et al. Prevalence of every night snoring and obstructive sleep apnoeas among 30-69-year-old men in Bologna, Italy. Acta Neurol Scand. 1989;79(5):366–72. doi: 10.1111/j.1600-0404.1989.tb03802.x 2741668

[62] Ferini-Strambi L, Zucconi M, Castronovo V, Garancini P, Oldani A, Smirne S. Snoring & sleep apnea: a population study in Italian women. Sleep. 1999;22(7):859–64. 10566904

[63] Ferini-Strambi L, Fantini ML, Castronovo C. Epidemiology of obstructive sleep apnea syndrome. Minerva Med. 2004;95(3):187–202. 15289748

[64] Benjafield AV, Ayas NT, Eastwood PR, Heinzer R, Ip MSM, Morrell MJ, et al. Estimation of the global prevalence and burden of obstructive sleep apnoea: a literature-based analysis. Lancet Respir Med. 2019;7(8):687–98. doi: 10.1016/S2213-2600(19)30198-5 31300334 PMC7007763

[65] Hedner J, Grote L, Bonsignore M, McNicholas W, Lavie P, Parati G, et al. The European Sleep Apnoea Database (ESADA): report from 22 European sleep laboratories. Eur Respir J. 2011;38(3):635–42. doi: 10.1183/09031936.00046710 21622583

[66] Xie C, Zhu R, Tian Y, Wang K. Association of obstructive sleep apnoea with the risk of vascular outcomes and all-cause mortality: a meta-analysis. BMJ Open. 2017;7(12):e013983. doi: 10.1136/bmjopen-2016-013983 29275335 PMC5770910

[67] Zhang XB, Peng LH, Lyu Z, Jiang XT, Du YP. Obstructive sleep apnoea and the incidence and mortality of cancer: a meta-analysis. Eur J Cancer Care (Engl). 2017;26(2). doi: 10.1111/ecc.12427 26660307

[68] Palamaner Subash Shantha G, Kumar AA, Cheskin LJ, Pancholy SB. Association between sleep-disordered breathing, obstructive sleep apnea, and cancer incidence: a systematic review and meta-analysis. Sleep Med. 2015;16(10):1289–94. doi: 10.1016/j.sleep.2015.04.014 26212231

[69] Zhu Z, Zhang F, Liu Y, Yang S, Li C, Niu Q, et al. Relationship of Obstructive Sleep Apnoea with Diabetic Retinopathy: A Meta-Analysis. Biomed Res Int. 2017;2017:4737064. doi: 10.1155/2017/4737064 29230409 PMC5694589

[70] Leong WB, Jadhakhan F, Taheri S, Thomas GN, Adab P. The Association between Obstructive Sleep Apnea on Diabetic Kidney Disease: A Systematic Review and Meta-Analysis. Sleep. 2016;39(2):301–8. doi: 10.5665/sleep.5432 26414891 PMC4712397

[71] Wang X, Bi Y, Zhang Q, Pan F. Obstructive sleep apnoea and the risk of type 2 diabetes: a meta-analysis of prospective cohort studies. Respirology. 2013;18(1):140–6. doi: 10.1111/j.1440-1843.2012.02267.x 22988888

[72] Xu S, Wan Y, Xu M, Ming J, Xing Y, An F, et al. The association between obstructive sleep apnea and metabolic syndrome: a systematic review and meta-analysis. BMC Pulm Med. 2015;15:105. doi: 10.1186/s12890-015-0102-3 26391008 PMC4578823

[73] Edwards C, Almeida OP, Ford AH. Obstructive sleep apnea and depression: A systematic review and meta-analysis. Maturitas. 2020;142:45–54. Epub 2020/11/08. doi: 10.1016/j.maturitas.2020.06.002 33158487

[74] Liu L, Kang R, Zhao S, Zhang T, Zhu W, Li E, et al. Sexual Dysfunction in Patients with Obstructive Sleep Apnea: A Systematic Review and Meta-Analysis. J Sex Med. 2015;12(10):1992–2003. doi: 10.1111/jsm.12983 26395783

[75] Sun AP, Liu N, Zhang YS, Zhao HY, Liu XL. The relationship between obstructive sleep apnea and Parkinson’s disease: a systematic review and meta-analysis. Neurol Sci. 2020;41(5):1153–62. Epub 2020/01/04. doi: 10.1007/s10072-019-04211-9 31897944

[76] Bakhsheshian J, Hwang MS, Friedman M. Association Between Obstructive Sleep Apnea and Spontaneous Cerebrospinal Fluid Leaks: A Systematic Review and Meta-analysis. JAMA Otolaryngol Head Neck Surg. 2015;141(8):733–8. doi: 10.1001/jamaoto.2015.1128 26110561

[77] Huon LK, Liu SY, Camacho M, Guilleminault C. The association between ophthalmologic diseases and obstructive sleep apnea: a systematic review and meta-analysis. Sleep Breath. 2016;20(4):1145–54. doi: 10.1007/s11325-016-1358-4 27230013

[78] Wu Y, Zhou LM, Lou H, Cheng JW, Wei RL. The Association Between Obstructive Sleep Apnea and Nonarteritic Anterior Ischemic Optic Neuropathy: A Systematic Review and Meta-Analysis. Curr Eye Res. 2016;41(7):987–92. doi: 10.3109/02713683.2015.1075221 26443989

[79] Hou H, Zhao Y, Yu W, Dong H, Xue X, Ding J, et al. Association of obstructive sleep apnea with hypertension: A systematic review and meta-analysis. J Glob Health. 2018;8(1):010405. doi: 10.7189/jogh.08.010405 29497502 PMC5825975

[80] Zhou X, Liu F, Zhang W, Wang G, Guo D, Fu W, et al. Obstructive sleep apnea and risk of aortic dissection: A meta-analysis of observational studies. Vascular. 2018;26(5):515–23. doi: 10.1177/1708538118766102 29566589

[81] Cao Y, Wu S, Zhang L, Yang Y, Cao S, Li Q. Association of allergic rhinitis with obstructive sleep apnea: A meta-analysis. Medicine (Baltimore). 2018;97(51):e13783. Epub 2018/12/24. doi: 10.1097/MD.0000000000013783 30572534 PMC6319794

[82] Musso G, Cassader M, Olivetti C, Rosina F, Carbone G, Gambino R. Association of obstructive sleep apnoea with the presence and severity of non-alcoholic fatty liver disease. A systematic review and meta-analysis. Obes Rev. 2013;14(5):417–31. doi: 10.1111/obr.12020 23387384

[83] Wu ZH, Yang XP, Niu X, Xiao XY, Chen X. The relationship between obstructive sleep apnea hypopnea syndrome and gastroesophageal reflux disease: a meta-analysis. Sleep Breath. 2018. doi: 10.1007/s11325-018-1691-x 29987514 PMC6529388

[84] Shi T, Min M, Sun C, Cheng C, Zhang Y, Liang M, et al. A meta-analysis of the association between gout, serum uric acid level, and obstructive sleep apnea. Sleep Breath. 2019;23(4):1047–57. Epub 2019/03/25. doi: 10.1007/s11325-019-01827-1 30903565

[85] Liu L, Su G, Wang S, Zhu B. The prevalence of obstructive sleep apnea and its association with pregnancy-related health outcomes: a systematic review and meta-analysis. Sleep Breath. 2019;23(2):399–412. Epub 2018/09/27. doi: 10.1007/s11325-018-1714-7 30255484

[86] Xu T, Feng Y, Peng H, Guo D, Li T. Obstructive sleep apnea and the risk of perinatal outcomes: a meta-analysis of cohort studies. Sci Rep. 2014;4:6982. doi: 10.1038/srep06982 25382105 PMC4225536

[87] Tregear S, Reston J, Schoelles K, Phillips B. Obstructive sleep apnea and risk of motor vehicle crash: systematic review and meta-analysis. J Clin Sleep Med. 2009;5(6):573–81. 20465027 PMC2792976

[88] Garbarino S, Guglielmi O, Sanna A, Mancardi GL, Magnavita N. Risk of Occupational Accidents in Workers with Obstructive Sleep Apnea: Systematic Review and Meta-analysis. Sleep. 2016;39(6):1211–8. doi: 10.5665/sleep.5834 26951401 PMC4863208

[89] Associazione Italiana di Oncologia Medica—Associazione Italiana dei Registri Tumori (AIOM-AIRTUM). I numeri del cancro in Italia 2018. http://www.registri-tumori.it/PDF/AIOM2017/2017_numeri_del_cancro.pdf.

[90] AMD, ANAAO-ASSOMED, Consorzio Mario Negri Sud, FAND-AID, FIMMG, Gruppo di Studio Complicanze Oculari della Società Italiana di Diabetologia, et al. Linee-guida per lo screening, la diagnostica e il trattamento della retinopatia diabetica in Italia 2015. https://www.fondazionebietti.it/sites/default/files/pdf/lg-rd-16sett2015.pdf.

[91] Associazione Medici Diabetologi—Società Italiana di Diabetologia (AMD-SID). Standard italiani per la cura del diabete mellito 2018. https://www.siditalia.it/pdf/Standard%20di%20Cura%20AMD%20-%20SID%202018_protetto2.pdf.

[92] International Diabetes Federation (IDF). IDF Diabetes Atlas, 8th edition. 2017.

[93] Tocci G, Ferrucci A, Bruno G, Mannarino E, Nati G, Trimarco B, et al. Prevalence of metabolic syndrome in the clinical practice of general medicine in Italy. Cardiovasc Diagn Ther. 2015;5(4):271–9. doi: 10.3978/j.issn.2223-3652.2015.07.03 26331111 PMC4536471

[94] Istituto nazionale di statistica (Istat). La salute mentale nelle fasi della vita 2018. https://www.istat.it/it/archivio/219807.

[95] Nicolosi A, Moreira ED Jr., Shirai M, Bin Mohd Tambi MI, Glasser DB. Epidemiology of erectile dysfunction in four countries: cross-national study of the prevalence and correlates of erectile dysfunction. Urology. 2003;61(1):201–6. doi: 10.1016/s0090-4295(02)02102-7 12559296

[96] Graziottin A. Prevalence and evaluation of sexual health problems—HSDD in Europe. J Sex Med. 2007;4 Suppl 3:211–9. doi: 10.1111/j.1743-6109.2007.00447.x 17394593

[97] Riccò M, Vezzosi L, Balzarini F, Gualerzi G, Ranzieri S, Signorelli C, et al. Prevalence of Parkinson Disease in Italy: a systematic review and meta-analysis. Acta Biomed. 2020;91(3):e2020088. doi: 10.23750/abm.v91i3.9443 32921784 PMC7717000

[98] Stevens E, Emmett E, Wang Y, McKevitt C, Wolfe C. The Burden of Stroke in Europe. Stroke Alliance for Europe. 2017.

[99] Tham YC, Li X, Wong TY, Quigley HA, Aung T, Cheng CY. Global prevalence of glaucoma and projections of glaucoma burden through 2040: a systematic review and meta-analysis. Ophthalmology. 2014;121(11):2081–90. doi: 10.1016/j.ophtha.2014.05.013 24974815

[100] Giampaoli S, Palmieri L, Donfrancesco C, Lo Noce C, Pilotto L, Vanuzzo D, et al. Cardiovascular health in Italy. Ten-year surveillance of cardiovascular diseases and risk factors: Osservatorio Epidemiologico Cardiovascolare/Health Examination Survey 1998–2012. Eur J Prev Cardiol. 2015;22(2 Suppl):9–37. doi: 10.1177/2047487315589011 26195612

[101] Dovellini EV. Percorso diagnostico dei pazienti ipertesi. Protocolli per l’ipertensione secondaria. Ital Heart J. 2000;1 (Suppl 5):53–9.

[102] Pacini D, Leone A, Belotti LM, Fortuna D, Gabbieri D, Zussa C, et al. Acute type A aortic dissection: significance of multiorgan malperfusion. Eur J Cardiothorac Surg. 2013;43(4):820–6. doi: 10.1093/ejcts/ezs500 23137559

[103] Younossi ZM, Blissett D, Blissett R, Henry L, Stepanova M, Younossi Y, et al. The economic and clinical burden of nonalcoholic fatty liver disease in the United States and Europe. Hepatology. 2016;64(5):1577–86. doi: 10.1002/hep.28785 27543837

[104] Darba J, Kaskens L, Plans P, Elizalde JI, Coma M, Cuomo R, et al. Epidemiology and societal costs of gastroesophageal reflux disease and Barrett’s syndrome in Germany, Italy and Spain. Expert Rev Pharmacoecon Outcomes Res. 2011;11(2):225–32. doi: 10.1586/erp.11.5 21476824

[105] Fox A, McHugh S, Browne J, Kenny LC, Fitzgerald A, Khashan AS, et al. Estimating the Cost of Preeclampsia in the Healthcare System: Cross-Sectional Study Using Data From SCOPE Study (Screening for Pregnancy End Points). Hypertension. 2017;70(6):1243–9. doi: 10.1161/HYPERTENSIONAHA.117.09499 29084880

[106] International Federation of Gynecology and Obstetrics (FIGO). Epidemiology of the hypertensive disorders of pregnancy. In: Magee L, von Dadelszen P, Stones W, Mathai M, editors. The FIGO Textbook of Pregnancy Hypertension: The Global Library of Women’s Medicine; 2016.

[107] Meregaglia M, Dainelli L, Banks H, Benedetto C, Detzel P, Fattore G. The short-term economic burden of gestational diabetes mellitus in Italy. BMC Pregnancy Childbirth. 2018;18(1):58. doi: 10.1186/s12884-018-1689-1 29471802 PMC5824573

[108] Merinopoulou E, Pokras S, Pimenta JM, Blini V, Veronesi C, Buda S, et al. The cost of preterm labor and preterm birth for mothers with uncomplicated pregnancies and their infants in Italy: a retrospective cohort study. Expert Rev Pharmacoecon Outcomes Res. 2018;19(2):231–41. doi: 10.1080/14737167.2018.1476340 29764243

[109] OECD. OECD Statistics. Health care use 2019 [Last access: 22nd March 2019]. https://stats.oecd.org.

[110] Istituto nazionale di statistica—Automobile Club d’Italia (Istat-Aci). La statistica ISTAT-ACI—Incidenti stradali 2017. http://www.aci.it/laci/studi-e-ricerche/dati-e-statistiche/incidentalita/la-statistica-istat-aci/2017.html.

[111] Fattore G, Torbica A, Susi A, Giovanni A, Benelli G, Gozzo M, et al. The social and economic burden of stroke survivors in Italy: a prospective, incidence-based, multi-centre cost of illness study. BMC Neurol. 2012;12:137. doi: 10.1186/1471-2377-12-137 23150894 PMC3536660

[112] Goldmeier D, Malik F, Phillips R, Green J. Cost implications of sexual dysfunction: the female picture. Int J Impot Res. 2004;16(2):130–4. doi: 10.1038/sj.ijir.3901179 14961049

[113] Happich M, Reitberger U, Breitscheidel L, Ulbig M, Watkins J. The economic burden of diabetic retinopathy in Germany in 2002. Graefes Arch Clin Exp Ophthalmol. 2008;246(1):151–9. doi: 10.1007/s00417-007-0573-x 17406883

[114] Koleva D, Motterlini N, Schiavone M, Garattini L, Study Group G. Medical costs of glaucoma and ocular hypertension in Italian referral centres: a prospective study. Ophthalmologica. 2007;221(5):340–7. doi: 10.1159/000104765 17728557

[115] Law A, McCoy M, Lynen R, Curkendall SM, Gatwood J, Juneau PL, et al. The prevalence of complications and healthcare costs during pregnancy. J Med Econ. 2015;18(7):533–41. doi: 10.3111/13696998.2015.1016229 25714263

[116] Leal J, Luengo-Fernandez R, Gray A, Petersen S, Rayner M. Economic burden of cardiovascular diseases in the enlarged European Union. Eur Heart J. 2006;27(13):1610–9. doi: 10.1093/eurheartj/ehi733 16495286

[117] Lucioni C, Mazzi S, Cerra C, Lottaroli S. I costi della sindrome metabolica. PharmacoEconomics Italian Research Articles. 2005;7(2):89–99. doi: 10.1007/bf03320540

[118] Luebke T, Brunkwall J. Cost-effectiveness of endovascular versus open repair of acute complicated type B aortic dissections. J Vasc Surg. 2014;59(5):1247–55. doi: 10.1016/j.jvs.2013.11.086 24418638

[119] Luengo-Fernandez R, Leal J, Gray A, Sullivan R. Economic burden of cancer across the European Union: a population-based cost analysis. Lancet Oncol. 2013;14(12):1165–74. doi: 10.1016/S1470-2045(13)70442-X 24131614

[120] Marcellusi A, Viti R, Mecozzi A, Mennini FS. The direct and indirect cost of diabetes in Italy: a prevalence probabilistic approach. Eur J Health Econ. 2016;17(2):139–47. doi: 10.1007/s10198-014-0660-y 25427540

[121] Mennini FS, Marcellusi A, von der Schulenburg JM, Gray A, Levy P, Sciattella P, et al. Cost of poor adherence to anti-hypertensive therapy in five European countries. Eur J Health Econ. 2015;16(1):65–72. doi: 10.1007/s10198-013-0554-4 24390212

[122] Olesen J, Gustavsson A, Svensson M, Wittchen HU, Jonsson B, group Cs, et al. The economic cost of brain disorders in Europe. Eur J Neurol. 2012;19(1):155–62. doi: 10.1111/j.1468-1331.2011.03590.x 22175760

[123] Institute of Medicine (US) Committee on Understanding Premature Birth and Assuring Healthy Outcomes. Preterm Birth: Causes, Consequences, and Prevention. Behrman RE, Butler AS, editors. Washington (DC)2007. 20669423

[124] Pizzo E. An estimate of costs and benefits of alternative methods of delivery: an empirical analysis. 2011.

[125] Romero-Aroca P, de la Riva-Fernandez S, Valls-Mateu A, Sagarra-Alamo R, Moreno-Ribas A, Soler N, et al. Cost of diabetic retinopathy and macular oedema in a population, an eight year follow up. BMC Ophthalmol. 2016;16:136. doi: 10.1186/s12886-016-0318-x 27491545 PMC4973531

[126] Schultz AB, Edington DW. Metabolic syndrome in a workplace: prevalence, co-morbidities, and economic impact. Metab Syndr Relat Disord. 2009;7(5):459–68. doi: 10.1089/met.2009.0008 19450154

[127] Wijnen W, Weijermars W, Vanden Berghe W, Schoeters A, Bauer R, Carnis L, et al. Crash cost estimates for European countries, Deliverable 3.2 of the H2020 project SafetyCube. 2017.

[128] Wilson EC, McKeen ES, Scuffham PA, Brown MC, Wylie K, Hackett G. The cost to the United Kingdom National Health Service of managing erectile dysfunction: the impact of sildenafil and prescribing restrictions. Pharmacoeconomics. 2002;20(13):879–89. doi: 10.2165/00019053-200220130-00002 12381240

[129] Zhou Z, Chaudhari P, Yang H, Fang AP, Zhao J, Law EH, et al. Healthcare Resource Use, Costs, and Disease Progression Associated with Diabetic Nephropathy in Adults with Type 2 Diabetes: A Retrospective Observational Study. Diabetes Ther. 2017;8(3):555–71. doi: 10.1007/s13300-017-0256-5 28361464 PMC5446382

[130] Armeni P, Bertolani A, Borsoi L, Costa F. La spesa sanitaria: composizione ed evoluzione. In: CERGAS, editor. Rapporto OASI 2018. Milano: Egea; 2018.

[131] Catala R, Villoro R, Merino M, Sangenis S, Colomes L, Hernandez Flix S, et al. Cost-effectiveness of Continuous Positive Airway Pressure Treatment in Moderate-Severe Obstructive Sleep Apnea Syndrome. Arch Bronconeumol. 2016;52(9):461–9. doi: 10.1016/j.arbres.2016.02.005 26993090

[132] Finkel KJ, Searleman AC, Tymkew H, Tanaka CY, Saager L, Safer-Zadeh E, et al. Prevalence of undiagnosed obstructive sleep apnea among adult surgical patients in an academic medical center. Sleep Med. 2009;10(7):753–8. doi: 10.1016/j.sleep.2008.08.007 19186102

[133] Simpson L, Hillman DR, Cooper MN, Ward KL, Hunter M, Cullen S, et al. High prevalence of undiagnosed obstructive sleep apnoea in the general population and methods for screening for representative controls. Sleep Breath. 2013;17(3):967–73. doi: 10.1007/s11325-012-0785-0 23161476

[134] Sia CH, Hong Y, Tan LWL, van Dam RM, Lee CH, Tan A. Awareness and knowledge of obstructive sleep apnea among the general population. Sleep Med. 2017;36:10–7. doi: 10.1016/j.sleep.2017.03.030 28735905

[135] Stores G. Clinical diagnosis and misdiagnosis of sleep disorders. J Neurol Neurosurg Psychiatry. 2007;78(12):1293–7. doi: 10.1136/jnnp.2006.111179 18024690 PMC2095611

[136] Flemons WW, Douglas NJ, Kuna ST, Rodenstein DO, Wheatley J. Access to diagnosis and treatment of patients with suspected sleep apnea. Am J Respir Crit Care Med. 2004;169(6):668–72. doi: 10.1164/rccm.200308-1124PP 15003950

[137] Weaver TE, Sawyer AM. Adherence to continuous positive airway pressure treatment for obstructive sleep apnoea: implications for future interventions. Indian J Med Res. 2010;131:245–58. 20308750 PMC2972705

[138] Weaver TE, Grunstein RR. Adherence to continuous positive airway pressure therapy: the challenge to effective treatment. Proc Am Thorac Soc. 2008;5(2):173–8. doi: 10.1513/pats.200708-119MG 18250209 PMC2645251

[139] Walter RJ, Hagedorn SI, Lettieri CJ. Impact of diagnosing and treating obstructive sleep apnea on healthcare utilization. Sleep Med. 2017;38:73–7. doi: 10.1016/j.sleep.2017.07.020 29031760

[140] Fu Y, Xia Y, Yi H, Xu H, Guan J, Yin S. Meta-analysis of all-cause and cardiovascular mortality in obstructive sleep apnea with or without continuous positive airway pressure treatment. Sleep Breath. 2017;21(1):181–9. doi: 10.1007/s11325-016-1393-1 27502205

[141] Kim Y, Koo YS, Lee HY, Lee SY. Can Continuous Positive Airway Pressure Reduce the Risk of Stroke in Obstructive Sleep Apnea Patients? A Systematic Review and Meta-Analysis. PLoS One. 2016;11(1):e0146317. doi: 10.1371/journal.pone.0146317 26731604 PMC4701420

[142] Antonopoulos CN, Sergentanis TN, Daskalopoulou SS, Petridou ET. Nasal continuous positive airway pressure (nCPAP) treatment for obstructive sleep apnea, road traffic accidents and driving simulator performance: a meta-analysis. Sleep Med Rev. 2011;15(5):301–10. doi: 10.1016/j.smrv.2010.10.002 21195643

[143] Tregear S, Reston J, Schoelles K, Phillips B. Continuous positive airway pressure reduces risk of motor vehicle crash among drivers with obstructive sleep apnea: systematic review and meta-analysis. Sleep. 2010;33(10):1373–80. doi: 10.1093/sleep/33.10.1373 21061860 PMC2941424

