# Supplementary material for: The invisible costs of obstructive sleep apnea (OSA): Systematic review and cost-of-illness analysis
Source: PLoS One. 2022 May 20;17(5):e0268677. doi: 10.1371/journal.pone.0268677 (PMC9122203; doi:10.1371/journal.pone.0268677)
Supplement: S1 Table — (DOCX) [file pone.0268677.s001.docx]

**S1 Table. Search strategy**

| **Database** | **Search strategy** | **Date of search** |
| --- | --- | --- |
| PubMed | 1. (associated[Title/Abstract] OR association[Title/Abstract]) OR (related[Title/Abstract] OR relation[Title/Abstract]) OR (connected[Title/Abstract] OR connection[Title/Abstract]) OR impos*[Title/Abstract] OR (correlated[Title/Abstract] OR correlation[Title/Abstract]) OR contribut*[Title/Abstract] OR impact*[Title/Abstract] OR cause*[Title/Abstract] OR afflict*[Title/Abstract] OR (risk factor[Title/Abstract] OR risk factors[Title/Abstract]) OR (odds ratio[Title/Abstract] OR odds ratios[Title/Abstract]) OR (effect[Title/Abstract] OR effects[Title/Abstract]) OR (consequence[Title/Abstract] OR consequences[Title/Abstract]) OR(comorbidity OR comorbidities[Title/Abstract] OR co-morbidity[Title/Abstract] OR co-morbidities[Title/Abstract]) OR (complication[Title/Abstract] OR complications[Title/Abstract]) 2. Obstructive Sleep Apnea[Title] OR Obstructive Sleep Apnoea[Title] OR OSA[Title] OR OSAS[Title] OR OSAHS[Title] OR Sleep Apnea, Obstructive[MeSH Major Topic] 3. #1 AND #2   Filters: Meta-Analysis, Systematic Reviews, Abstract, Humans, English. | First search: November 19^th^, 2018 |
| PubMed | 1. (associated[Title/Abstract] OR association[Title/Abstract]) OR (related[Title/Abstract] OR relation[Title/Abstract]) OR (connected[Title/Abstract] OR connection[Title/Abstract]) OR impos*[Title/Abstract] OR (correlated[Title/Abstract] OR correlation[Title/Abstract]) OR contribut*[Title/Abstract] OR impact*[Title/Abstract] OR cause*[Title/Abstract] OR afflict*[Title/Abstract] OR (risk factor[Title/Abstract] OR risk factors[Title/Abstract]) OR (odds ratio[Title/Abstract] OR odds ratios[Title/Abstract]) OR (effect[Title/Abstract] OR effects[Title/Abstract]) OR (consequence[Title/Abstract] OR consequences[Title/Abstract]) OR(comorbidity OR comorbidities[Title/Abstract] OR co-morbidity[Title/Abstract] OR co-morbidities[Title/Abstract]) OR (complication[Title/Abstract] OR complications[Title/Abstract]) 2. Obstructive Sleep Apnea[Title] OR Obstructive Sleep Apnoea[Title] OR OSA[Title] OR OSAS[Title] OR OSAHS[Title] OR Sleep Apnea, Obstructive[MeSH Major Topic] 3. 2018/11/20:2021/05/13[dp] 4. #1 AND #2 AND #3   Filters: Meta-Analysis, Systematic Reviews, Abstract, Humans, English. | Updated search: May 13^th^, 2021 |
