# Supplementary material for: The invisible costs of obstructive sleep apnea (OSA): Systematic review and cost-of-illness analysis
Source: PLoS One. 2022 May 20;17(5):e0268677. doi: 10.1371/journal.pone.0268677 (PMC9122203; doi:10.1371/journal.pone.0268677)
Supplement: S2 Table — (DOCX) [file pone.0268677.s002.docx]

**S2 Table. Exclusion criteria**

| **Exclusion criteria** | |
| --- | --- |
| **Titles and abstracts** | - Focus on animals. - Reverse association only, i.e. condition as risk factor for OSA. - Complete absence of information about possible association of OSA with other clinical or non-clinical conditions. - Only association with biomarkers, metabolites, genes or proteins. - Focus on children. - Focus on complications or operative outcomes. - Focus on symptoms or physiological states. - Focus on a very specific population (e.g. indigenous). - Type of study different from systematic reviews and meta-analyses. |
