# Supplementary material for: The invisible costs of obstructive sleep apnea (OSA): Systematic review and cost-of-illness analysis
Source: PLoS One. 2022 May 20;17(5):e0268677. doi: 10.1371/journal.pone.0268677 (PMC9122203; doi:10.1371/journal.pone.0268677)
Supplement: S1 File — (DOCX) [file pone.0268677.s003.docx]

**S3 File. Additional information on OSA prevalence estimation**

**Estimates from the population-based study**

Considering the data from the HypnoLaus study [[1](#_ENREF_1)] and using the distribution of patients between moderate and severe OSA provided by Hedner and colleagues [[2](#_ENREF_2)], we derived prevalence rates for population aged 40-85 years, as shown in the table below. Using data from Istat on the resident population in Italy in 2018 [[3](#_ENREF_3)] and the prevalence rates, we computed prevalence in absolute terms, i.e. the number of people in Italy aged 40-85 years with OSA.

|  | **Female** | **Male** | **Total** |
| --- | --- | --- | --- |
| ***Rates*** | | | |
| Mild (5≤AHI<15) | 37.4% | 34.1% |  |
| Moderate-severe (AHI≥15) | 23.4% | 49.7% |  |
| Moderate (15≤AHI<30) | 12.5% | 19.9% |  |
| Severe (AHI≥30) | 10.9% | 29.8% |  |
| Overall (AHI≥5) | 60.8% | 83.8% |  |
| ***Absolute values*** | | | |
| Mild (5≤AHI<15) | 6,703,067 | 5,582,051 | 12,285,118 |
| Moderate-severe (AHI≥15) | 4,193,897 | 8,135,717 | 12,329,614 |
| Moderate (15≤AHI<30) | 2,236,745 | 3,260,161 | 5,496,906 |
| Severe (AHI≥30) | 1,957,152 | 4,875,556 | 6,832,708 |
| Overall (AHI≥5) | 10,896,964 | 13,717,768 | 24,614,732 |

*Source. Rates: Our elaboration from Hedner et al (2011) and Heinzer et al (2015). Absolute values: Our elaboration using computed prevalence rates and ISTAT data on Italian resident population aged 40-85 in 2018.*

From the prevalence in absolute terms provided in the table above, we estimated the prevalence rates for the general adult population (aged 15-74 years), reported in the main text. It is important to underline that in computing these rates we made a conservative assumption, i.e. that the prevalence between 15 and 39 years old is equal to zero.

**Estimates from the literature-based study**

The study by Benjafield et al [[4](#_ENREF_4)] provided prevalence estimates for the Italian total population aged 30-69 years, without sex stratification. Using these total prevalence data, the female/male split obtained from the HypnoLaus study [[1](#_ENREF_1)] and the distribution of patients between moderate and severe OSA provided by Hedner and colleagues [[2](#_ENREF_2)], we derived prevalence rates for population aged 30-69 years, as shown in the table below. Using data from Istat on the resident population in Italy in 2018 [[3](#_ENREF_3)] and the prevalence rates, we computed prevalence in absolute terms, i.e. the number of people in Italy aged 40-85 years with OSA.

|  | **Female** | **Male** | **Total** |
| --- | --- | --- | --- |
| ***Rates*** | | | |
| Mild (5≤AHI<15) | 9.8% | 7.1% |  |
| Moderate-severe (AHI≥15) | 8.0% | 16.1% |  |
| Moderate (15≤AHI<30) | 4.3% | 6.4% |  |
| Severe (AHI≥30) | 3.7% | 9.6% |  |
| Overall (AHI≥5) | 17.9% | 23.2% |  |
| ***Absolute values*** | | | |
| Mild (5≤AHI<15) | 1,657,025 | 1,163,534 | 2,820,559 |
| Moderate-severe (AHI≥15) | 1,354,459 | 2,627,507 | 3,981,966 |
| Moderate (15≤AHI<30) | 722,378 | 1,052,900 | 1,775,278 |
| Severe (AHI≥30) | 632,081 | 1,574,607 | 2,206,688 |
| Overall (AHI≥5) | 3,011,484 | 3,791,041 | 6,802,526 |

From the prevalence in absolute terms provided in the table above, we estimated the prevalence rates for the general adult population (aged 15-74 years), reported in the main text. As above, it is important to underline that in computing these rates we made a conservative assumption, i.e. that the prevalence for the population not considered in the study by Benjafield et al [[4](#_ENREF_4)] (between 15-29 and 70-74 years old) is equal to zero.

# References

1. Heinzer R, Vat S, Marques-Vidal P, Marti-Soler H, Andries D, Tobback N, et al. Prevalence of sleep-disordered breathing in the general population: the HypnoLaus study. Lancet Respir Med. 2015;3(4):310-8. doi: 10.1016/S2213-2600(15)00043-0.

2. Hedner J, Grote L, Bonsignore M, McNicholas W, Lavie P, Parati G, et al. The European Sleep Apnoea Database (ESADA): report from 22 European sleep laboratories. Eur Respir J. 2011;38(3):635-42. doi: 10.1183/09031936.00046710.

3. Istituto nazionale di statistica (Istat). Statistiche Istat [Last access: 9th April 2019]. Available from: <http://dati.istat.it/>.

4. Benjafield AV, Ayas NT, Eastwood PR, Heinzer R, Ip MSM, Morrell MJ, et al. Estimation of the global prevalence and burden of obstructive sleep apnoea: a literature-based analysis. Lancet Respir Med. 2019;7(8):687-98. doi: 10.1016/S2213-2600(19)30198-5.
