# Supplementary material for: The invisible costs of obstructive sleep apnea (OSA): Systematic review and cost-of-illness analysis
Source: PLoS One. 2022 May 20;17(5):e0268677. doi: 10.1371/journal.pone.0268677 (PMC9122203; doi:10.1371/journal.pone.0268677)
Supplement: S3 File — (DOCX) [file pone.0268677.s005.docx]

**S5 File. Prevalence of conditions significantly associated with OSA**

| **Condition** | **Prevalence (adult population aged 15-74)** | | **Source** |
| --- | --- | --- | --- |
|  | **Absolute value** | **Rate** |  |
| All-cause mortality | 148,527 | 0.3% | Istat [[1](#_ENREF_1)] |
| Cardiovascular mortality | 32,471 | 0.1% | Istat [[1](#_ENREF_1)] |
| Cancer^^ | 1,890,000 | 4.2% | AIOM-AIRTUM (2018) [[2](#_ENREF_2)] |
| Diabetic retinopathy | 1,191,175 | 2.6% | AMD et al (2015) [[3](#_ENREF_3)] |
| Diabetic kidney disease | 688,541 | 1.5% | AMD-SID (2018) [[4](#_ENREF_4)] IDF (2017) [[5](#_ENREF_5)] |
| Type 2 diabetes | 3,098,432 | 6.8% | IDF (2017) [[5](#_ENREF_5)] |
| Metabolic syndrome | 14,948,577 | 33.0% | Tocci et al (2015) [[6](#_ENREF_6)] |
| Depression^†^ | 724,779 | 1.6% | Istat (2018) [[7](#_ENREF_7)] |
| Erectile dysfunction^‡^ | 2,243,158 | 10.0% | Nicolosi et al (2003) [[8](#_ENREF_8)] |
| Female sexual dysfunction^‡‡^ | 6,653,836 | 29.0% | Graziottin (2007) [[9](#_ENREF_9)] |
| Parkinson's disease | 56,229 | 0.1% | Riccò et al (2020) [[10](#_ENREF_10)] |
| Stroke^∫^ | 73,116 | 0.2% | Stevens et al (2017) [[11](#_ENREF_11)] |
| Glaucoma^¥^ | 811,685 | 1.8% | Tham et al (2014) [[12](#_ENREF_12)] |
| Resistant hypertension | 722,517 | 1.6% | Giampaoli et al (2015) [[13](#_ENREF_13)] Dovellini (2000) [[14](#_ENREF_14)] |
| Essential hypertension | 13,727,826 | 30.3% | Giampaoli et al (2015) [[13](#_ENREF_13)] Dovellini (2000) [[14](#_ENREF_14)] |
| Ischemic heart disease | 2,276,838 | 5.0% | Giampaoli et al (2015) [[13](#_ENREF_13)] |
| Aortic dissection^∫^ | 1,609 | 0.004% | Pacini et al (2013) [[15](#_ENREF_15)] |
| Non-alcoholic fatty liver disease | 9,285,722 | 20.5% | Younossi et al (2016) [[16](#_ENREF_16)] |
| Gastroesophageal reflux disease | 4,892,262 | 10.8% | Darbà et al (2011) [[17](#_ENREF_17)] |
| Pre-eclampsia^‡‡,Δ^ | 9,163 | 0.04% | Fox et al (2017) [[18](#_ENREF_18)] |
| Gestational hypertension^‡‡,ΔΔ^ | 13,745 | 0.06% | FIGO (2016) [[19](#_ENREF_19)] |
| Gestational diabetes ^‡‡^ | 49,938 | 0.22% | Meregaglia et al (2018) [[20](#_ENREF_20)] |
| Preterm delivery^‡‡^ | 27,947 | 0.1% | Merinopoulou et al (2018) [[21](#_ENREF_21)] |
| Cesarean delivery^‡‡^ | 165,440 | 0.7% | OECD [[22](#_ENREF_22)] |
| Car accidents | 217,096 | 0.5% | Istat-Aci (2017) [[23](#_ENREF_23)] |
| Work accidents* | 25,587 | 0.1% | Istat-Aci (2017) [[23](#_ENREF_23)] |

*Note.* ^^*Prevalence of breast, colorectal, prostate, lung and central nervous system cancers. ^†^Estimates are referred to major depression. ^‡^The reference population for the calculation of prevalence rate is Italian male population. ^‡‡^The reference population for the calculation of prevalence rate is Italian female population.* ^¥^*We used European prevalence data. ^∫^Incidence data were considered.* ^Δ^ *Irish prevalence data were used.* ^ΔΔ^*Worldwide prevalence data were used. *We considered the number of commercial motor vehicle crashes as the studies included in Garbarino et al (2016) are mostly focused on these work-related accidents.*

# References

1. Istituto nazionale di statistica (Istat). Statistiche Istat [Last access: 9th April 2019]. Available from: <http://dati.istat.it/>.

2. Associazione Italiana di Oncologia Medica - Associazione Italiana dei Registri Tumori (AIOM-AIRTUM). I numeri del cancro in Italia 2018. Available from: <http://www.registri-tumori.it/PDF/AIOM2017/2017_numeri_del_cancro.pdf>.

3. AMD, ANAAO-ASSOMED, Consorzio Mario Negri Sud, FAND-AID, FIMMG, Gruppo di Studio Complicanze Oculari della Società Italiana di Diabetologia, et al. Linee-guida per lo screening, la diagnostica e il trattamento della retinopatia diabetica in Italia 2015. Available from: https://[www.fondazionebietti.it/sites/default/files/pdf/lg-rd-16sett2015.pdf](http://www.fondazionebietti.it/sites/default/files/pdf/lg-rd-16sett2015.pdf).

4. Associazione Medici Diabetologi - Società Italiana di Diabetologia (AMD-SID). Standard italiani per la cura del diabete mellito 2018. Available from: https://[www.siditalia.it/pdf/Standard%20di%20Cura%20AMD%20-%20SID%202018_protetto2.pdf](http://www.siditalia.it/pdf/Standard%20di%20Cura%20AMD%20-%20SID%202018_protetto2.pdf).

5. IDF International Diabetes Federation. IDF Diabetes Atlas, 8th edition. 2017.

6. Tocci G, Ferrucci A, Bruno G, Mannarino E, Nati G, Trimarco B, et al. Prevalence of metabolic syndrome in the clinical practice of general medicine in Italy. Cardiovasc Diagn Ther. 2015;5(4):271-9. doi: 10.3978/j.issn.2223-3652.2015.07.03.

7. Istituto nazionale di statistica (Istat). La salute mentale nelle fasi della vita 2018. Available from: https://[www.istat.it/it/archivio/219807](http://www.istat.it/it/archivio/219807).

8. Nicolosi A, Moreira ED, Jr., Shirai M, Bin Mohd Tambi MI, Glasser DB. Epidemiology of erectile dysfunction in four countries: cross-national study of the prevalence and correlates of erectile dysfunction. Urology. 2003;61(1):201-6.

9. Graziottin A. Prevalence and evaluation of sexual health problems--HSDD in Europe. J Sex Med. 2007;4 Suppl 3:211-9. doi: 10.1111/j.1743-6109.2007.00447.x.

10. Riccò M, Vezzosi L, Balzarini F, Gualerzi G, Ranzieri S, Signorelli C, et al. Prevalence of Parkinson Disease in Italy: a systematic review and meta-analysis. Acta Biomed. 2020;91(3):e2020088. doi: 10.23750/abm.v91i3.9443.

11. Stevens E, Emmett E, Wang Y, McKevitt C, Wolfe C. The Burden of Stroke in Europe. Stroke Alliance for Europe. 2017.

12. Tham YC, Li X, Wong TY, Quigley HA, Aung T, Cheng CY. Global prevalence of glaucoma and projections of glaucoma burden through 2040: a systematic review and meta-analysis. Ophthalmology. 2014;121(11):2081-90. doi: 10.1016/j.ophtha.2014.05.013.

13. Giampaoli S, Palmieri L, Donfrancesco C, Lo Noce C, Pilotto L, Vanuzzo D, et al. Cardiovascular health in Italy. Ten-year surveillance of cardiovascular diseases and risk factors: Osservatorio Epidemiologico Cardiovascolare/Health Examination Survey 1998-2012. Eur J Prev Cardiol. 2015;22(2 Suppl):9-37. doi: 10.1177/2047487315589011.

14. Dovellini EV. Percorso diagnostico dei pazienti ipertesi. Protocolli per l’ipertensione secondaria. Ital Heart J. 2000;1 (Suppl 5):53-9.

15. Pacini D, Leone A, Belotti LM, Fortuna D, Gabbieri D, Zussa C, et al. Acute type A aortic dissection: significance of multiorgan malperfusion. Eur J Cardiothorac Surg. 2013;43(4):820-6. doi: 10.1093/ejcts/ezs500.

16. Younossi ZM, Blissett D, Blissett R, Henry L, Stepanova M, Younossi Y, et al. The economic and clinical burden of nonalcoholic fatty liver disease in the United States and Europe. Hepatology. 2016;64(5):1577-86. doi: 10.1002/hep.28785.

17. Darba J, Kaskens L, Plans P, Elizalde JI, Coma M, Cuomo R, et al. Epidemiology and societal costs of gastroesophageal reflux disease and Barrett's syndrome in Germany, Italy and Spain. Expert Rev Pharmacoecon Outcomes Res. 2011;11(2):225-32. doi: 10.1586/erp.11.5.

18. Fox A, McHugh S, Browne J, Kenny LC, Fitzgerald A, Khashan AS, et al. Estimating the Cost of Preeclampsia in the Healthcare System: Cross-Sectional Study Using Data From SCOPE Study (Screening for Pregnancy End Points). Hypertension. 2017;70(6):1243-9. doi: 10.1161/HYPERTENSIONAHA.117.09499.

19. International Federation of Gynecology and Obstetrics (FIGO). Epidemiology of the hypertensive disorders of pregnancy. In: Magee L, von Dadelszen P, Stones W, Mathai M, editors. The FIGO Textbook of Pregnancy Hypertension: The Global Library of Women’s Medicine; 2016.

20. Meregaglia M, Dainelli L, Banks H, Benedetto C, Detzel P, Fattore G. The short-term economic burden of gestational diabetes mellitus in Italy. BMC Pregnancy Childbirth. 2018;18(1):58. doi: 10.1186/s12884-018-1689-1.

21. Merinopoulou E, Pokras S, Pimenta JM, Blini V, Veronesi C, Buda S, et al. The cost of preterm labor and preterm birth for mothers with uncomplicated pregnancies and their infants in Italy: a retrospective cohort study. Expert Rev Pharmacoecon Outcomes Res. 2018;19(2):231-41. doi: 10.1080/14737167.2018.1476340.

22. OECD. OECD.Stat [Last access: 9th April 2019]. Available from: https://stats.oecd.org/.

23. Istituto nazionale di statistica - Automobile Club d'Italia (Istat-Aci). La statistica ISTAT-ACI - Incidenti stradali 2017. Available from: <http://www.aci.it/laci/studi-e-ricerche/dati-e-statistiche/incidentalita/la-statistica-istat-aci/2017.html>.
