# Supplementary material for: The invisible costs of obstructive sleep apnea (OSA): Systematic review and cost-of-illness analysis
Source: PLoS One. 2022 May 20;17(5):e0268677. doi: 10.1371/journal.pone.0268677 (PMC9122203; doi:10.1371/journal.pone.0268677)
Supplement: S4 File — (DOCX) [file pone.0268677.s006.docx]

**S6 File. Indirect costs due to all-cause and cardiovascular mortality influenced by OSA**

In order to consider the burden of premature death associated with OSA, we estimated productivity costs through the friction method [[1](#_ENREF_1)]. Productivity costs were estimated for different age groups to account for differences in wages. Age and gender-specific yearly paid production values were drawn from Pradelli and Ghetti (2017) [[2](#_ENREF_2)]. Employment rates^[[1]](#footnote-1)^ and all-cause mortality data for 2018 (last year available) were retrieved from Istat [[3](#_ENREF_3)]. PAF estimates for both model 1 and model 2 were used to identify the number of deaths due to OSA in each age group (in absence of more detailed data, we assumed that the PAF was equal across all age groups). An overview of data used is provided in the table below.

| **Age** | **Model 1** | | | **Model 2** | | | **Employment rate** | | **Annual paid production value** | |
| --- | --- | --- | --- | --- | --- | --- | --- | --- | --- | --- |
|  | **# deaths associated with OSA** | | | **# deaths associated with OSA** | | |  |  |  |  |
|  | **Male** | **Female** | **Total** | **Male** | **Female** | **Total** | **Male** | **Female** | **Male** | **Female** |
| 15-19 | 27 | 10 | 37 | 16 | 6 | 22 | 27 | 14.3% | € 6,465 | € 3,766 |
| 20-24 | 40 | 16 | 56 | 24 | 10 | 34 | 40 | 14.3% | € 7,539 | € 4,368 |
| 25-29 | 46 | 18 | 64 | 28 | 11 | 39 | 46 | 53.3% | € 24,001 | € 11,847 |
| 30-34 | 57 | 27 | 84 | 34 | 16 | 51 | 57 | 53.3% | € 30,587 | € 14,820 |
| 35-39 | 85 | 48 | 133 | 51 | 29 | 80 | 85 | 62.6% | € 30,587 | € 14,820 |
| 40-44 | 173 | 100 | 273 | 104 | 60 | 164 | 173 | 62.6% | € 36,498 | € 17,172 |
| 45-49 | 305 | 189 | 494 | 183 | 114 | 297 | 305 | 60.8% | € 27,768 | € 11,512 |
| 50-54 | 501 | 310 | 811 | 301 | 186 | 487 | 501 | 60.8% | € 32,088 | € 13,293 |
| 55-59 | 732 | 436 | 1168 | 440 | 262 | 702 | 732 | 43.9% | € 32,088 | € 13,293 |
| 60-64 | 1067 | 621 | 1688 | 641 | 373 | 1014 | 1067 | 43.9% | € 39,829 | € 15,221 |
| 65-69 | 1647 | 983 | 2630 | 989 | 591 | 1580 | 1647 | 0.0% | € 3,168 | € 507 |
| 70-74 | 2254 | 1437 | 3691 | 1354 | 863 | 2217 | 2254 | 0.0% | € 3,168 | € 507 |

*Note. Model 1: statistics calculated using OSA prevalence data derived from the population-based study. Model 2: statistics calculated using OSA prevalence data derived from the literature-based study.*

Using a friction period of 75 days for all age groups, we computed productivity losses due to all-cause mortality associated with OSA. Moreover, using data on cardiovascular mortality and the corresponding PAF, we estimated the portion of overall mortality productivity losses due to cardiovascular diseases associated with OSA.

| **Age** | **Model 1** | | **Model 2** | |
| --- | --- | --- | --- | --- |
|  | **Indirect costs due to all-cause mortality influenced by OSA** | **Indirect costs due to cardiovascular mortality influenced by OSA** | **Indirect costs due to all-cause mortality influenced by OSA** | **Indirect costs due to cardiovascular mortality influenced by OSA** |
| 15-19 | € 8,541 | € 2,063 | € 2,914 | € 789 |
| 20-24 | € 14,925 | € 3,400 | € 5,093 | € 1,301 |
| 25-29 | € 182,432 | € 55,222 | € 62,247 | € 21,134 |
| 30-34 | € 295,315 | € 134,575 | € 100,763 | € 51,503 |
| 35-39 | € 541,676 | € 324,025 | € 184,823 | € 124,006 |
| 40-44 | € 1,311,257 | € 830,687 | € 447,408 | € 317,909 |
| 45-49 | € 1,734,865 | € 1,156,147 | € 591,945 | € 442,465 |
| 50-54 | € 3,293,065 | € 2,285,437 | € 1,123,611 | € 874,652 |
| 55-59 | € 3,623,670 | € 2,519,023 | € 1,236,416 | € 964,047 |
| 60-64 | € 6,462,568 | € 4,452,966 | € 2,205,063 | € 1,704,180 |
| 65-74 | € 0 | € 0 | € 0 | € 0 |
| **Total** | **€ 17,468,314** | **€ 11,763,544** | **€ 5,960,283** | **€ 4,501,987** |

*Note. Model 1: statistics calculated using OSA prevalence data derived from the population-based study. Model 2: statistics calculated using OSA prevalence data derived from the literature-based study.*

# References

1. Koopmanschap MA, van Ineveld BM. Towards a new approach for estimating indirect costs of disease. Soc Sci Med. 1992;34(9):1005-10. doi: 10.1016/0277-9536(92)90131-9.

2. Pradelli L, Ghetti G. A general model for the estimation of societal costs of lost production and informal care in Italy. 2017. 2017;18(1). Epub 2017-02-21. doi: 10.7175/fe.v18i1.1278.

3. Istituto nazionale di statistica (Istat). Statistiche Istat [Last access: 9th April 2019]. Available from: <http://dati.istat.it/>.

1. Employment rates are available until age 64, therefore we assumed an employment rate equal to 0% for people aged 65-74 years. [↑](#footnote-ref-1)
