# Supplementary material for: The invisible costs of obstructive sleep apnea (OSA): Systematic review and cost-of-illness analysis
Source: PLoS One. 2022 May 20;17(5):e0268677. doi: 10.1371/journal.pone.0268677 (PMC9122203; doi:10.1371/journal.pone.0268677)
Supplement: S5 File — (DOCX) [file pone.0268677.s007.docx]

**S7 File. Cost of conditions (cost per patient/year of conditions associated with OSA - 2018 inflation and PPP adjusted)**

| **Condition** | **Mean annual cost per patient, adjusted for inflation and PPP** | | | | **Source of original cost data** |
| --- | --- | --- | --- | --- | --- |
|  | **Direct healthcare cost** | **Direct non-healthcare cost** | **Productivity losses cost*** | **Total cost** |  |
| Cancer | € 5,718 | € 4,581 | € 119 | € 10,418 | Luengo-Fernandez et al (2013) [[1](#_ENREF_1)] |
| Diabetic retinopathy | € 307 | € 242 | € 579 | € 1,128 | Romero-Aroca et al (2016) [[2](#_ENREF_2)] Happich et al (2008) [[3](#_ENREF_3)] |
| Diabetic kidney disease | € 797 |  |  | € 797 | Zhou et al (2017) [[4](#_ENREF_4)] |
| Type 2 diabetes | € 3,866 |  | € 4,352 | € 8,217 | Marcellusi et al (2016) [[5](#_ENREF_5)] |
| Metabolic syndrome | € 1,900 |  | € 90 | € 1,990 | Lucioni et al (2005) [[6](#_ENREF_6)] Schultz et al (2009) [[7](#_ENREF_7)] |
| Depression^^ | € 871 | € 496 | € 1,947 | € 3,313 | Olesen et al (2012) [[8](#_ENREF_8)] |
| Erectile dysfunction | € 407 |  |  | € 407 | Wilson et al (2002) [[9](#_ENREF_9)] |
| Female sexual dysfunction | € 761 |  |  | € 761 | Goldmeier et al (2004) [[10](#_ENREF_10)] |
| Parkinson's disease | € 6,146 | € 4,825 | € 1,212 | € 12,183 | Olesen et al (2012) [[8](#_ENREF_8)] |
| Stroke | € 13,452 | € 8,490 | € 907 | € 22,848 | Fattore et al (2012) [[11](#_ENREF_11)] |
| Glaucoma | € 985 |  |  | € 985 | Koleva et al (2007) [[12](#_ENREF_12)] |
| Resistant hypertension | € 239 |  |  | € 239 | Mennini et al (2015) [[13](#_ENREF_13)] |
| Essential hypertension | € 239 |  |  | € 239 | Mennini et al (2015) [[13](#_ENREF_13)] |
| Ischemic heart disease | € 1,496 | € 348 | € 458 | € 2,303 | Leal et al (2006) [[14](#_ENREF_14)] |
| Aortic dissection | € 38,064 |  |  | € 38,064 | Luebke et al (2014) [[15](#_ENREF_15)] |
| Non-alcoholic fatty liver disease ¥ | € 1,197 |  | € 4,424 | € 5,622 | Younossi et al (2016) [[16](#_ENREF_16)] |
| Gastroesophageal reflux disease | € 308 |  | € 186 | € 494 | Darbà et al (2011) [[17](#_ENREF_17)] |
| Pre-eclampsia | € 4,668 |  |  | € 4,668 | Fox et al (2017) [[18](#_ENREF_18)] |
| Gestational hypertension^Δ^ | € 11,856 |  |  | € 11,856 | Law et al (2015) [[19](#_ENREF_19)] |
| Gestational diabetes | € 3,755 |  |  | € 3,755 | Meregaglia et al (2017) [[20](#_ENREF_20)] |
| Preterm delivery^Δ^ | € 9,025 |  | € 9,782 | € 18,807 | Merinopoulou et al (2018) [[21](#_ENREF_21)] Institute of Medicine (2007) [[22](#_ENREF_22)] |
| Cesarean delivery ¥¥ | € 2,515 |  | € 943 | € 3,458 | Pizzo (2011) [[23](#_ENREF_23)] |
| Car accidents † | € 9,348 |  | € 23,834 | € 33,182 | Wijnen et al (2017) [[24](#_ENREF_24)] |
| Work accidents †‡ | € 9,348 |  | € 23,834 | € 33,182 | Wijnen et al (2017) [[24](#_ENREF_24)] |

*Note. *Only productivity losses due to morbidity were included when the original study reported separate estimates for costs due morbidity and mortality.* ^^*Costs are referred to major depression.* ^Δ^ *We adopted a conservative approach and considered costs related to uncomplicated pregnancies.* ¥ *These costs were calculated by estimating the annual quality-adjusted life-years (QALYs) lost due to NAFLD and by applying a monetary value to this QALY estimate. ¥¥ Also direct non-healthcare costs are included in this estimate, but unfortunately from the data provided in the study it was not possible to isolate them. †We adopted a conservative approach and considered only costs due to serious and slight injuries, excluding costs due to fatal crashes. ‡We considered costs due to motor vehicle accidents as the studies included in Garbarino et al (2016) are mostly focused on commercial motor vehicle crashes.*

# References

1. Luengo-Fernandez R, Leal J, Gray A, Sullivan R. Economic burden of cancer across the European Union: a population-based cost analysis. The Lancet Oncology. 2013;14(12):1165-74. doi: 10.1016/S1470-2045(13)70442-X. PubMed PMID: 24131614.

2. Romero-Aroca P, de la Riva-Fernandez S, Valls-Mateu A, Sagarra-Alamo R, Moreno-Ribas A, Soler N, et al. Cost of diabetic retinopathy and macular oedema in a population, an eight year follow up. BMC ophthalmology. 2016;16:136. doi: 10.1186/s12886-016-0318-x. PubMed PMID: 27491545; PubMed Central PMCID: PMC4973531.

3. Happich M, Reitberger U, Breitscheidel L, Ulbig M, Watkins J. The economic burden of diabetic retinopathy in Germany in 2002. Graefe's archive for clinical and experimental ophthalmology = Albrecht von Graefes Archiv fur klinische und experimentelle Ophthalmologie. 2008;246(1):151-9. doi: 10.1007/s00417-007-0573-x. PubMed PMID: 17406883.

4. Zhou Z, Chaudhari P, Yang H, Fang AP, Zhao J, Law EH, et al. Healthcare Resource Use, Costs, and Disease Progression Associated with Diabetic Nephropathy in Adults with Type 2 Diabetes: A Retrospective Observational Study. Diabetes therapy : research, treatment and education of diabetes and related disorders. 2017;8(3):555-71. doi: 10.1007/s13300-017-0256-5. PubMed PMID: 28361464; PubMed Central PMCID: PMC5446382.

5. Marcellusi A, Viti R, Mecozzi A, Mennini FS. The direct and indirect cost of diabetes in Italy: a prevalence probabilistic approach. The European journal of health economics : HEPAC : health economics in prevention and care. 2016;17(2):139-47. doi: 10.1007/s10198-014-0660-y. PubMed PMID: 25427540.

6. Lucioni C, Mazzi S, Cerra C, Lottaroli S. I costi della sindrome metabolica. PharmacoEconomics Italian Research Articles. 2005;7(2):89-99. doi: 10.1007/bf03320540.

7. Schultz AB, Edington DW. Metabolic syndrome in a workplace: prevalence, co-morbidities, and economic impact. Metabolic syndrome and related disorders. 2009;7(5):459-68. doi: 10.1089/met.2009.0008. PubMed PMID: 19450154.

8. Olesen J, Gustavsson A, Svensson M, Wittchen HU, Jonsson B, group Cs, et al. The economic cost of brain disorders in Europe. European journal of neurology. 2012;19(1):155-62. doi: 10.1111/j.1468-1331.2011.03590.x. PubMed PMID: 22175760.

9. Wilson EC, McKeen ES, Scuffham PA, Brown MC, Wylie K, Hackett G. The cost to the United Kingdom National Health Service of managing erectile dysfunction: the impact of sildenafil and prescribing restrictions. PharmacoEconomics. 2002;20(13):879-89. doi: 10.2165/00019053-200220130-00002. PubMed PMID: 12381240.

10. Goldmeier D, Malik F, Phillips R, Green J. Cost implications of sexual dysfunction: the female picture. International journal of impotence research. 2004;16(2):130-4. doi: 10.1038/sj.ijir.3901179. PubMed PMID: 14961049.

11. Fattore G, Torbica A, Susi A, Giovanni A, Benelli G, Gozzo M, et al. The social and economic burden of stroke survivors in Italy: a prospective, incidence-based, multi-centre cost of illness study. BMC neurology. 2012;12:137. doi: 10.1186/1471-2377-12-137. PubMed PMID: 23150894; PubMed Central PMCID: PMC3536660.

12. Koleva D, Motterlini N, Schiavone M, Garattini L, Study Group G. Medical costs of glaucoma and ocular hypertension in Italian referral centres: a prospective study. Ophthalmologica Journal international d'ophtalmologie International journal of ophthalmology Zeitschrift fur Augenheilkunde. 2007;221(5):340-7. doi: 10.1159/000104765. PubMed PMID: 17728557.

13. Mennini FS, Marcellusi A, von der Schulenburg JM, Gray A, Levy P, Sciattella P, et al. Cost of poor adherence to anti-hypertensive therapy in five European countries. The European journal of health economics : HEPAC : health economics in prevention and care. 2015;16(1):65-72. doi: 10.1007/s10198-013-0554-4. PubMed PMID: 24390212.

14. Leal J, Luengo-Fernandez R, Gray A, Petersen S, Rayner M. Economic burden of cardiovascular diseases in the enlarged European Union. European heart journal. 2006;27(13):1610-9. doi: 10.1093/eurheartj/ehi733. PubMed PMID: 16495286.

15. Luebke T, Brunkwall J. Cost-effectiveness of endovascular versus open repair of acute complicated type B aortic dissections. Journal of vascular surgery. 2014;59(5):1247-55. doi: 10.1016/j.jvs.2013.11.086. PubMed PMID: 24418638.

16. Younossi ZM, Blissett D, Blissett R, Henry L, Stepanova M, Younossi Y, et al. The economic and clinical burden of nonalcoholic fatty liver disease in the United States and Europe. Hepatology. 2016;64(5):1577-86. doi: 10.1002/hep.28785. PubMed PMID: 27543837.

17. Darba J, Kaskens L, Plans P, Elizalde JI, Coma M, Cuomo R, et al. Epidemiology and societal costs of gastroesophageal reflux disease and Barrett's syndrome in Germany, Italy and Spain. Expert review of pharmacoeconomics & outcomes research. 2011;11(2):225-32. doi: 10.1586/erp.11.5. PubMed PMID: 21476824.

18. Fox A, McHugh S, Browne J, Kenny LC, Fitzgerald A, Khashan AS, et al. Estimating the Cost of Preeclampsia in the Healthcare System: Cross-Sectional Study Using Data From SCOPE Study (Screening for Pregnancy End Points). Hypertension. 2017;70(6):1243-9. doi: 10.1161/HYPERTENSIONAHA.117.09499. PubMed PMID: 29084880.

19. Law A, McCoy M, Lynen R, Curkendall SM, Gatwood J, Juneau PL, et al. The prevalence of complications and healthcare costs during pregnancy. Journal of medical economics. 2015;18(7):533-41. doi: 10.3111/13696998.2015.1016229. PubMed PMID: 25714263.

20. Meregaglia M, Dainelli L, Banks H, Benedetto C, Detzel P, Fattore G. The short-term economic burden of gestational diabetes mellitus in Italy. BMC pregnancy and childbirth. 2018;18(1):58. doi: 10.1186/s12884-018-1689-1. PubMed PMID: 29471802; PubMed Central PMCID: PMC5824573.

21. Merinopoulou E, Pokras S, Pimenta JM, Blini V, Veronesi C, Buda S, et al. The cost of preterm labor and preterm birth for mothers with uncomplicated pregnancies and their infants in Italy: a retrospective cohort study. Expert review of pharmacoeconomics & outcomes research. 2018;19(2):231-41. doi: 10.1080/14737167.2018.1476340. PubMed PMID: 29764243.

22. Outcomes IoMUCoUPBaAH. Preterm Birth: Causes, Consequences, and Prevention. Behrman RE, Butler AS, editors. Washington (DC)2007.

23. Pizzo E. An estimate of costs and benefits of alternative methods of delivery: an empirical analysis. 2011.

24. Wijnen W, Weijermars W, Vanden Berghe W, Schoeters A, Bauer R, Carnis L, et al. Crash cost estimates for European countries, Deliverable 3.2 of the H2020 project SafetyCube. 2017.
