# Supplementary material for: The invisible costs of obstructive sleep apnea (OSA): Systematic review and cost-of-illness analysis
Source: PLoS One. 2022 May 20;17(5):e0268677. doi: 10.1371/journal.pone.0268677 (PMC9122203; doi:10.1371/journal.pone.0268677)
Supplement: S6 File — (DOCX) [file pone.0268677.s008.docx]

**S8 File. Cost of OSA diagnosis and treatment**

According to a study conducted by the ESADA (European Sleep Apnoea Database) group and expert opinion, in Italy the current clinical practice for OSA’s diagnosis entails both cardio-respiratory polygraphy (CRPG) and polysomnography (PSG) [[1](#_ENREF_1)]. The golden standard for objective assessment of OSA is in-hospital, technician-attended polysomnography (PSG). However, the use of this diagnostic test is limited by its high cost and limited accessibility to sleep centres. Therefore, outpatient portable PSG and CRPG are often used. Unfortunately, there are no studies published on peer-reviewed journals providing data on the cost of different diagnostic pathway for OSA in Italy, so we relied on different sources. Based on the data reported in a cost-effectiveness analysis conducted by Català and colleagues [[2](#_ENREF_2)], we assumed that OSA diagnosis is performed by standard PSG monitored in the hospital in 32.4% of cases. In the remaining cases (67.6%), we assumed that half of the patients undergo outpatient PSG and CRPG respectively, in both cases after an initial visit with a specialist (e.g. neurologist). For in-hospital PSG costs, we considered the data provided by a preliminary study conducted by the Sleep Centre of San Raffaele hospital (Milan, Italy) and published on an Italian newspaper (Sanità24 - Il Sole 24 Ore). The cost for the first visit with a specialist is already included in the costs provided. The cost for inpatient diagnosis can be either covered by NHS or paid by the patient. On the basis of a recent estimate published by the Observatory on Healthcare Organizations and Policies in Italy (OASI) [[3](#_ENREF_3)], we assumed that costs are covered by the NHS in the 74% of cases. For outpatient diagnostic pathway, we considered the unit cost (tariff) for diagnostic exams (i.e. PSG and CRPG) and for a consultation with a specialist retrieved from official Italian sources [[4](#_ENREF_4)]. **Fig 1** shows the distribution of patients according to different diagnostic pathways, while **Table 1** provides a summary of the unit cost for OSA diagnosis in Italy.

Considering patients’ distribution across diagnostic pathways and the costs associated with them, OSA diagnosis costs amount to approximately €381 per patient.

Fig 1 Distribution of patients by diagnostic pathway

Table 1 OSA(S) diagnostic tests: unit cost in Italy

| **Diagnostic test** | | **Unit cost** | **Source** |
| --- | --- | --- | --- |
| Outpatient | Initial visit with a specialist | €20.66 | Nomenclatore dell’assistenza specialistica ambulatoriale |
|  | Polysomnography | €139.44 |  |
|  | Polygraphy | €51.13 |  |
| Inpatient | Polysomnography covered by NHS | €880 | Sanità24 - Il Sole 24 Ore |
|  | Polysomnography not covered by NHS | €1090 |  |

In the absence of published data, we estimated the cost of treating an OSA patient with CPAP (the most diffused treatment) using the cost data provided by a CPAP producer and supplier (Philips S.p.A.). In Italy the device is usually rented by the National Health Service (NHS), at a cost of approximately €0.70 per day, and this cost is covered by the NHS in the 60% of cases. We assumed that the cost was the same for patients for whom the NHS does not cover CPAP cost.

Therefore, the cost per treated patient amounts to approximately €256 per year (€0.70 * 365 days).

# References

1. Fietze I, Penzel T, Alonderis A, Barbe F, Bonsignore MR, Calverly P, et al. Management of obstructive sleep apnea in Europe. Sleep Med. 2011;12(2):190-7. doi: 10.1016/j.sleep.2010.10.003.

2. Catala R, Villoro R, Merino M, Sangenis S, Colomes L, Hernandez Flix S, et al. Cost-effectiveness of Continuous Positive Airway Pressure Treatment in Moderate-Severe Obstructive Sleep Apnea Syndrome. Arch Bronconeumol. 2016;52(9):461-9. doi: 10.1016/j.arbres.2016.02.005.

3. Del Vecchio M, Fenech L, Rappini V. I consumi privati in sanità. In: CERGAS, editor. Rapporto OASI 2018. Milano: Egea; 2018.

4. Nomenclatore dell’assistenza specialistica ambulatoriale [Last access: 15th April 2019]. Available from: <http://www.salute.gov.it/portale/temi/p2_6.jsp?lingua=italiano&id=1767&area=programmazioneSanitariaLea&menu=lea>.
