# Supplementary material for: The invisible costs of obstructive sleep apnea (OSA): Systematic review and cost-of-illness analysis
Source: PLoS One. 2022 May 20;17(5):e0268677. doi: 10.1371/journal.pone.0268677 (PMC9122203; doi:10.1371/journal.pone.0268677)
Supplement: S7 File — (DOCX) [file pone.0268677.s009.docx]

**S9 File. Additional information on QALYs value lost calculation**

For QALYs value lost calculation, we used the utility values provided by Català and colleagues [[1](#_ENREF_1)], who conducted a study on OSA patients treated with CPAP with a before-after design. Although RCTs usually represent the best available evidence due to randomization and the presence of a control group, the two RCTs found through literature review present some limitations: the study by Chakravorty et al (2002) [[2](#_ENREF_2)] had a very small sample of patients (n=57) and a short follow-up (3 months); the study by McMillan et al (2014) [[3](#_ENREF_3)] focused on older patients (mean age=71) who are not representative of the patient population considered in the present report (aged 15-74). The selected study by Català and colleagues provides the most recent estimates on QoL, derived from a large sample of patients (n=373) with a sufficiently long follow-up (1 year). A possible drawback of this study is that it considered severe patients (mean AHI=54.3), therefore the health utility gain may be slightly overestimated. However, comparing these results with those provided by Mar et al (2003) [[4](#_ENREF_4)], who considered less severe patients (mean AHI=41.3) and estimated a utility gain of 0.07, we may conclude that the potential risk of overestimation is acceptable.

We estimated QALYs lost for both alive and dead untreated patients. For the latter, using data on the effect of CPAP treatment on mortality reduction [[5](#_ENREF_5)], we estimated the proportion of untreated patients among total dead OSA patients in one year (i.e. 11,229 and 3,797 for model 1 and model 2 respectively). Then, we hypothesized that, on average, the patients died in the middle of the year. We obtained the following estimates for model 1 and model 2 respectively:

Model 1

$${QALYs value lost}_{alive}=\left[ \left( 0.84-0.79 \right)*12,092,910 \right]*€14,860=€ 8,985,031,870$$

$${QALYs lost}_{dead}=\left[ \left( 0.84-\left( \frac{0.79}{2}+\frac{0.0}{2} \right) \right)*6,704 \right]*€14,860=€ 44,333,851$$

$${QALYs value lost}_{total}={QALYs value lost}_{alive}+{QALYs value lost}_{dead}=€9,029,365,722$$

Model 2

$${QALYs value lost}_{alive}=\left[ \left( 0.84-0.79 \right)*3,751,966 \right]*€14,860=€ 2,786,011,416$$

$${QALYs lost}_{dead}=\left[ \left( 0.84-\left( \frac{0.79}{2}+\frac{0.0}{2} \right) \right)*2,287 \right]*€14,860=€ 15,125,555$$

$${QALYs value lost}_{total}={QALYs value lost}_{alive}+{QALYs value lost}_{dead}=€ 2,801,136,971$$

# References

1. Catala R, Villoro R, Merino M, Sangenis S, Colomes L, Hernandez Flix S, et al. Cost-effectiveness of Continuous Positive Airway Pressure Treatment in Moderate-Severe Obstructive Sleep Apnea Syndrome. Arch Bronconeumol. 2016;52(9):461-9. doi: 10.1016/j.arbres.2016.02.005.

2. Chakravorty I, Cayton RM, Szczepura A. Health utilities in evaluating intervention in the sleep apnoea/hypopnoea syndrome. Eur Respir J. 2002;20(5):1233-8.

3. McMillan A, Bratton DJ, Faria R, Laskawiec-Szkonter M, Griffin S, Davies RJ, et al. A multicentre randomised controlled trial and economic evaluation of continuous positive airway pressure for the treatment of obstructive sleep apnoea syndrome in older people: PREDICT. Health Technol Assess. 2015;19(40):1-188. doi: 10.3310/hta19400.

4. Mar J, Rueda JR, Duran-Cantolla J, Schechter C, Chilcott J. The cost-effectiveness of nCPAP treatment in patients with moderate-to-severe obstructive sleep apnoea. Eur Respir J. 2003;21(3):515-22. doi: 10.1183/09031936.03.00040903.

5. Fu Y, Xia Y, Yi H, Xu H, Guan J, Yin S. Meta-analysis of all-cause and cardiovascular mortality in obstructive sleep apnea with or without continuous positive airway pressure treatment. Sleep Breath. 2017;21(1):181-9. doi: 10.1007/s11325-016-1393-1.
