# Supplementary material for: The invisible costs of obstructive sleep apnea (OSA): Systematic review and cost-of-illness analysis
Source: PLoS One. 2022 May 20;17(5):e0268677. doi: 10.1371/journal.pone.0268677 (PMC9122203; doi:10.1371/journal.pone.0268677)
Supplement: Attachment — Submitted filename: Response to Reviewers.docx [file pone.0268677.s010.docx]

Dear Academic Editor and Reviewers,

We would like to thank you for your valuable comments, which helped us to further improve the manuscript. Please find below our answers to each revision request.

Academic Editor

1. Please ensure that your manuscript meets PLOS ONE's style requirements, including those for file naming.

We thank the Editor for this comment. We have checked the entire manuscript and revised the title, citing the method used (i.e. systematic review).

2. We note that your literature search was performed on 2018;to allow an up-to-date view of the topic, we would request that the search is updated. Moreover, to meet our criteria on reproducibility, please provide more information on how data obtained from the systematic review was analysed and used for your cost- of-illness analysis.

We thank the Editor for this useful comment. We have updated the review as requested (last update: 13^th^ May 2021), revised the manuscript and all other materials (figures, supplementary files). Please, note that, besides the manuscript, the following files have been revised: Fig 1, Fig 2, Fig 3, S1 Table, S4 File, S5 File, S7 File. We reported the detail on how data obtained from the systematic review was analysed and used for cost-of-illness analysis in the main text. In particular, main information can be found in the methods section titled “Identification of conditions associated with OSA”.

3. We note that this manuscript is a systematic review or meta-analysis; our author guidelines therefore require that you use PRISMA guidance to help improve reporting quality of this type of study. Please upload copies of the completed PRISMA checklist as Supporting Information with a file name “PRISMA checklist”.

We thank the Editor for the comment. The PRISMA checklist had been already included in the original submission as supporting file (S4 File). However, we have updated it after the changes made to the manuscript.

4. Thank you for stating the following in the Financial Disclosure section:

CERGAS SDA Bocconi received an unrestricted grant for research from Philips S.p.A. The funder had no role in study design, data collection and analysis, decision to publish, or preparation of the manuscript.

We note that you received funding from a commercial source: Philips S.p.A

Please provide an amended Competing Interests Statement that explicitly states this commercial funder, along with any other relevant declarations relating to employment, consultancy, patents, products in development, marketed products, etc.

Within this Competing Interests Statement, please confirm that this does not alter your adherence to all PLOS ONE policies on sharing data and materials by including the following statement: "This does not alter our adherence to PLOS ONE policies on sharing data and materials.”

We thank the Editor for the comment. We have changed the Competing Interests Statement and reported the amended version in the cover letter as requested.

5. Thank you for stating the following in the Competing Interests section:

Ludovica Borsoi, Patrizio Armeni, Gleb Donin and Francesco Costa have no competing interests todeclare. Luigi Ferini-Strambi declares the following competing interests (last 3 years): Philips-Respironics (fee for lectures), Resmed (fee for advisory board).

Please confirm that this does not alter your adherence to all PLOS ONE policies on sharing data and materials, by including the following statement: "This does not alter our adherence to PLOS ONE policies on sharing data and materials.” (as detailed online in our guide for authorshttp://journals.plos.org/plosone/s/competing-interests). If there are restrictions on sharing of data and/or materials, please state these. Please note that we cannot proceed with consideration of your article until this information has been declared.

We thank the Editor for the comment. We have changed the Competing Interests Statement and reported the amended version in the cover letter as requested.

Reviewer #1

This study submitted by Borsoi and colleagues aimed at assessing the economic burden of OSA in the adult population in Italy. The attempt is interesting but there are several concerns regarding these data:

1. Literature review is by far non exhaustive.

We thank the Reviewer for the comment. We acknowledge the fact that our inclusion criteria are particularly strict, since we decided to include only systematic reviews and meta-analyses. The reason for this choice is that we were looking for directional associations between OSA and other conditions and realized that in most cases the literature was extremely heterogeneous in terms of methods, definitions and results: therefore we opted for the “systematic review of systematic reviews” (Smith et al., 2011) which is a recommended method in this kind of cases. Moreover, we further restricted the selection on systematic reviews presenting a meta-analysis because we needed a reliable quantitative assessment of the intensity of association between OSA and other conditions. We know that a vast literature exists, suggesting that OSA might be connected to many conditions, but in some cases there is no sufficient quantitative evidence and in other cases there is no quantitative evidence at all. We decided to keep a conservative approach. Our results show that, even based *only* on the most reliable quantitative evidence (systematic reviews with meta-analyses) the economic impact of OSA on the society is huge, well beyond the one suggested so far by less structured studies. In addition, with respect to the original manuscript, we have updated the literature review to 2021 in order to collect relevant evidence published after the original search carried out on November 19^th^, 2018. We have acknowledged in the “Study limitations” section that the review performed may not be fully exhaustive as we did not consider individual studies but focused only on systematic reviews and meta-analyses, including the latter for COI analysis and included the methodological reference suggesting the “systematic review of systematic reviews” approach. In this perspective, the aim of our literature review is to be exhaustive in systematic reviews and meta-analyses only.

2. The novelty is limited as previous similar analysis and markov models have been conducted and published both in scientific journals or reported by Frost and Sullivan and Mc Kinsey (“The price of fatigue”) for the American academy of sleep medicine.
3. The main novelty is to provide these data for Italy. I think that the paper is more suitable for an Italian journal of health policy or management.

We thank the Reviewer for these comments (2 and 3). We acknowledge the fact that other studies have been previously investigated the topic in other national contexts (we have cited the most relevant and comprehensive ones in the “Introduction” section). However, some of them either did not perform a systematic review to identify the conditions influenced by OSA (e.g., Hillman et al, 2018) or were not published in peer-reviewed journals (e.g., report by McKinsey) and in general all of them followed a less systematic and structured approach, thus not limiting the risk of biased analysis (e.g. the report by McKinsey was not transparent on the reason for inclusion of specific association measures between OSA and other conditions). We believe that, given the enormous amount of studies, but the relative scarcity of reliable synthetic quantitative information, a well structured, transparent and solid approach is necessary when investigating OSA’s relationship with other clinical and non-clinical conditions. Therefore, in addition to having adopted a systematic approach in the identification of studies reporting quantitative data on the influence of OSA on all other clinical and non-clinical conditions, the novelty of our study is that we included in our cost-of-illness analysis exclusively those providing the highest level of evidence, i.e. meta-analyses, overcoming the limitations of single studies. Moreover, unlike other studies, we provided also estimates on the QALYs value lost due to undertreatment of OSA. This is a major point, with strong policy implications, since it allows to measure not only the financial component of the cost-of-illness but also the economic counterpart of the health benefit lost by patients (which is the most important object of public expenditure in health). We show how important this component of the burden is, and no previous study did something similar. Although our results are focused on a specific national context (i.e., Italy), we believe that the methodological approach used, and extensively reported in our manuscript, may be interesting also for a non-Italian readership, also taking into account that all other studies are based on specific national contexts (e.g. Australia, the US, etc.). Differently from the previous literature, however, the specific context could be considered a limitation only with respect to epidemiological data, while our per-patient results are more generalizable than the ones presented in previous studies since we based our analysis on synthetic evidence and not on single studies.

Reviewer #2

1. I think the topic is of outmost importance. The problem is hugely undermined in daily life. Therefore the possible outcomes in socioeconomic domain is a smart one to explore. I only have one comment to the authors: We have seen in the last Covid outbreak that no tow country is same for medical measures. Therefore the sentence on line 136 "referred to other countries whose health care systems can be comparable to the Italian one" may actually be off.

We completely agree with the Reviewer’s point and revised the sentence. Moreover, we have also reported this limitation in a dedicated section (“Study limitations”). We thank the Reviewer for the useful comment.

Reviewer #3

1. Obstructive sleep apnoea (OSA) is an underdiagnosed chronic disease with a high prevalence in adults. As it is becoming a significant social problem associated with a low quality of life and increased mortality, the cost-effectiveness ratio of diagnostic and therapeutic management of OSA is important to counteract the demand of objective diagnosis. This cost-of-illness study is considered an essential evaluation technique in health care, helping health-care decision-makers to set up and prioritize health-care policies and interventions. Therefore, this well written manuscript is worth being published.

We thank the Reviewer for the positive and very kind feedback.
