# Supplementary material for: The invisible costs of obstructive sleep apnea (OSA): Systematic review and cost-of-illness analysis
Source: PLoS One. 2022 May 20;17(5):e0268677. doi: 10.1371/journal.pone.0268677 (PMC9122203; doi:10.1371/journal.pone.0268677)
Supplement: Attachment — Submitted filename: Response to Reviewers_round2.docx [file pone.0268677.s011.docx]

Dear Academic Editor and Reviewer,

Please find below our answers to your valuable comments.

Academic Editor

1. Based on the reviewer comments, which were quite different and based on the diversity of comments, in conclusion, we like to follow the more critical decision. May be a more specialized journal will appreciate more your specific work.

I am sorry that we cannot be more positive on this occasion, but hope that you appreciate the reasons for this decision.

Additional Editor Comments :

Dear authors, we acknowledge the large amount of data. This is exceptional. We also acknowledge the hard work provided. However regarding a cost-of-illness analysis, our knowledgeable reviewers remain to be very critical. Therefore we decided to reject the manuscript.

The Academic Editor's decision appears to be in contrast with the comments received by the majority of reviewers in both rounds of revision and is also manifestly inconsistent with the points on which the Editor asked the authors to intervene on. In fact, in the first round of revision (mainly favorable, as two of the reviewers outlined the importance of the topic, and one of them explicitly underlined that the manuscript was worth being published), the Editor asked us to update the systematic review in order to "allow an up-to-date view of the topic". Following the Editors' comment, we updated the literature review, that required a substantial work. However, in the second round of revision, despite having acknowledged "the large amount of data", "the hard work provided" and the rigor of the methods used (reviewer's comment: "Enormous amount of data, and hardworking, sophisticated approach to a very complex topic", "Well realized literature update, text, and data ameliorations", "methods are developed originally, and compiled in a concise way"), the Editor decided that the cost-of-illness was not of interest of the journal ("a more specialized journal will appreciate more your specific work"). Moreover, we would like to underline that PLOS ONE has published several cost-of-illness studies over the last years, and some of them have used methods similar to those presented in our paper. We include here a non-exhaustive but exemplary list of cost-of-illness analyses published on the journal, some of which are very recent:

- Armour, Mike, et al. "The cost of illness and economic burden of endometriosis and chronic pelvic pain in Australia: A national online survey." PloS one 14.10 (2019): e0223316.
- Curado, Daniel da Silva Pereira, et al. "Direct cost of systemic arterial hypertension and its complications in the circulatory system from the perspective of the Brazilian public health system in 2019." PloS one 16.6 (2021): e0253063.
- de Oliveira, Michele Lessa, Leonor Maria Pacheco Santos, and Everton Nunes da Silva. "Direct healthcare cost of obesity in Brazil: an application of the cost-of-illness method from the perspective of the public health system in 2011." PloS one 10.4 (2015): e0121160.
- Ernstsson, Olivia, et al. "Cost of illness of multiple sclerosis-a systematic review." PloS one 11.7 (2016): e0159129.
- Ilboudo, Patrick G., et al. "Cost-of-illness of cholera to households and health facilities in rural Malawi." PloS one 12.9 (2017): e0185041.
- Javanbakht, Mehdi, et al. "Cost-of-illness analysis of type 2 diabetes mellitus in Iran." PloS one 6.10 (2011): e26864.
- Jo, Minkyung, et al. "The cost-of-illness trend of schizophrenia in South Korea from 2006 to 2016." PloS one 15.7 (2020): e0235736.
- Matsumoto, Kunichika, et al. "Cost of illness of hepatocellular carcinoma in Japan: A time trend and future projections." Plos one 13.6 (2018): e0199188.
- Moran, Patrick S., et al. "Economic burden of maternal morbidity–A systematic review of cost-of-illness studies." PloS one 15.1 (2020): e0227377.
- Oliveira, Luana Nice da Silva, Alexander Itria, and Erika Coutinho Lima. "Cost of illness and program of dengue: A systematic review." PloS one 14.2 (2019): e0211401.
- Pares-Badell, Oleguer, et al. "Cost of disorders of the brain in Spain." PloS one 9.8 (2014): e105471.
- Steinke, Sabine IB, et al. "Cost-of-illness in psoriasis: comparing inpatient and outpatient therapy." PLoS One 8.10 (2013): e78152.

Based on the positive comments on the methodological quality and relevance of results and on the fact that this kind of studies has been often published in this journal, we believe that this manuscript could be relevant for PLOS ONE.

Reviewer #4:

1. Enormous amount of data, and hardworking, sophisticated approach to a very complex topic, revealing many levels of socio-economic approach to health economy.

Well realized literature update, text, and data ameliorations according to the first couple of reviews!

The country preference (Italy) in view of this reviewer seems of minor relevance, because data mainly rely on costs, and prevalence of diverse illnesses in relation to OSA. The latter are rather independent of country.

Thus the elaborate methods are developed originally, and compiled in a concise way, presented in relatively brief descriptions.

We thank the reviewer for the positive feedback.

2. The presentation of results mainly rely on the population attributable fraction (PAF), which is enhanced by use of numerous specific variables of health economy (OR, RR, HR, QALY, WTP, and different measures of OSA-probability). The reason for this elaborated development of PAF remains unclear (in the methods description). Moreover it is not discussed or critically reflected in the discussion/conclusions part.

These methods are an innovative approach to the complex topic of compiling the "invisible" (indirect) costs of OSA. Because of reasons of this intelligent, unique approach to specific topics of socio-economy, it becomes clear that the article does not completely fulfill the criteria of PLOS.

A journal of health economy seems appropriate for publication (e.g. Eur. J Health Econ., Health Economics, J Health Economics).

Their readers are suggested to be customized to the methodological approach.

Thus reviewer encourages the authors to publish in one of these lines of business.

We thank the reviewer for the comments. As regards the PAF, as explained in the methods section, we had to rely on different formulae as the measures of association of OSA with other conditions found in the meta-analyses included were heterogeneous (i.e., relative risk, odds ratio or hazard ratio) and required different approaches. In the manuscript, we have clearly specified the references we relied on in order to select the most appropriate formulae on the basis of association data.

As regards the adherence of our manuscript with the journal's scope, we would like to underline that PLOS ONE has published several cost-of-illness studies over the last years, and some of them have used methods similar to those presented in our paper. Therefore, we believe that our manuscript could be relevant for the journal and its audience. Please find below some examples of cost-of-illness studies published on PLOS ONE:

- Armour, Mike, et al. "The cost of illness and economic burden of endometriosis and chronic pelvic pain in Australia: A national online survey." PloS one 14.10 (2019): e0223316.
- Curado, Daniel da Silva Pereira, et al. "Direct cost of systemic arterial hypertension and its complications in the circulatory system from the perspective of the Brazilian public health system in 2019." PloS one 16.6 (2021): e0253063.
- de Oliveira, Michele Lessa, Leonor Maria Pacheco Santos, and Everton Nunes da Silva. "Direct healthcare cost of obesity in Brazil: an application of the cost-of-illness method from the perspective of the public health system in 2011." PloS one 10.4 (2015): e0121160.
- Ernstsson, Olivia, et al. "Cost of illness of multiple sclerosis-a systematic review." PloS one 11.7 (2016): e0159129.
- Ilboudo, Patrick G., et al. "Cost-of-illness of cholera to households and health facilities in rural Malawi." PloS one 12.9 (2017): e0185041.
- Javanbakht, Mehdi, et al. "Cost-of-illness analysis of type 2 diabetes mellitus in Iran." PloS one 6.10 (2011): e26864.
- Jo, Minkyung, et al. "The cost-of-illness trend of schizophrenia in South Korea from 2006 to 2016." PloS one 15.7 (2020): e0235736.
- Matsumoto, Kunichika, et al. "Cost of illness of hepatocellular carcinoma in Japan: A time trend and future projections." Plos one 13.6 (2018): e0199188.
- Moran, Patrick S., et al. "Economic burden of maternal morbidity–A systematic review of cost-of-illness studies." PloS one 15.1 (2020): e0227377.
- Oliveira, Luana Nice da Silva, Alexander Itria, and Erika Coutinho Lima. "Cost of illness and program of dengue: A systematic review." PloS one 14.2 (2019): e0211401.
- Pares-Badell, Oleguer, et al. "Cost of disorders of the brain in Spain." PloS one 9.8 (2014): e105471.
- Steinke, Sabine IB, et al. "Cost-of-illness in psoriasis: comparing inpatient and outpatient therapy." PLoS One 8.10 (2013): e78152.

3. With respect to the many additional S1-9 files it seems rather difficult to understand data processing completely.

In relation to this, the length of the manuscript is rather brief, but overcrowded by the many assumptions (partly from literature) to implement the various items and methodological techniques of health economy.

We thank the reviewer for the comments. The structure of our manuscript is the result of a clear and reasoned choice. In the main text, indeed, we provide information on the most relevant methodological aspects that allow the reader to understand the results presented. Through the supplementary materials, instead, we aimed at providing additional information on some methodological steps that, despite not being fundamental to understand the concepts discussed in the main text, are deemed useful in order to fully grasp the results obtained and ensure transparency of our work.

We acknowledge that we relied on some assumptions in order to develop our analyses, but we thoroughly justified and referenced them throughout the text.

4. Female sexual dysfunction for development of PAF data in OSA is well accepted. But what is the contribution of Caesarean delivery to PAF in OSA?

We thank the reviewer for the question. There are several studies, included in the meta-analysis by Liu et al (2019), which demonstrated that OSA is related to an increased risk for Caesarean delivery. Therefore, as our study aims at estimating the economic burden influenced by OSA, i.e. the proportion of the cost-of-illness of conditions for which OSA is a proven risk factor that can be attributed to OSA itself, we included this maternal-related outcome in our analyses.

5. General discussion: Aren't the variables used rather part of indirect costs instead of invisible (intangible) costs used in the title?

We thank the reviewer for the question. When we talk about the "invisible costs" of OSA in the title, we are not referring to a specific cost category (e.g. indirect costs), but rather to the fact that the burden generated by OSA is somehow hidden (thus "invisible") behind the cost of other conditions for which it is a risk factor.
